# Growing with dinosaurs: natural products from the Cretaceous relict *Metasequoia glyptostroboides* Hu & Cheng—a molecular reservoir from the ancient world with potential in modern medicine

**DOI:** 10.1007/s11101-015-9395-3

**Published:** 2015-02-22

**Authors:** Ole Johan Juvik, Xuan Hong Thy Nguyen, Heidi Lie Andersen, Torgils Fossen

**Affiliations:** 1 Department of Chemistry and Centre for Pharmacy, University of Bergen, Allégt. 41, 5007 Bergen, Norway; 2 University Museum of Bergen, University of Bergen, Thormøhlensgt. 53 A, 5007 Bergen, Norway

**Keywords:** *Metasequoia glyptostroboides*, Natural products, Biological activity, Paleobotany, Living fossil

## Abstract

After the sensational rediscovery of living exemplars of the Cretaceous relict *Metasequoia glyptostroboides*—a tree previously known exclusively from fossils from various locations in the northern hemisphere, there has been an increasing interest in discovery of novel natural products from this unique plant source. This article includes the first complete compilation of natural products reported from *M. glyptostroboides* during the entire period in which the tree has been investigated (1954–2014) with main focus on the compounds specific to this plant source. Studies on the biological activity of pure compounds and extracts derived from *M. glyptostroboides* are reviewed for the first time. The unique potential of *M. glyptostroboides* as a source of bioactive constituents is founded on the fact that the tree seems to have survived unchanged since the Cretaceous era. Since then, its molecular defense system has resisted the attacks of millions of generations of pathogens. In line with this, some recent landmarks in *Metasequoia* paleobotany are covered. Initial spectral analysis of recently discovered intact 53 million year old wood and amber of *Metasequoia* strongly indicate that the tree has remained unchanged for millions of years at the molecular level.

## Introduction

*Metasequoia glyptostroboides* Hu et Cheng (Cupressaceae) is a deciduous conifer native to southeast China (Hu 1948b). The tree is particularly interesting because it seems to have remained unchanged for millions of years since the Cretaceous period (145–66 million years ago). During this long timespan the tree has survived substantial ecological and climate changes and resisted attacks from countless generations of bacteria, viruses, fungi and other plant pathogens. Phytochemical investigations of natural products from *M. glyptostroboides* have been performed since the early 1950s (Bate-Smith 1954; Bate-Smith and Lerner 1954; Hattori et al. 1954). A significant number of natural products have hitherto been characterised from *M. glyptostroboides* although there is as yet no complete review of natural products thereof. In current literature a limited number of natural products from *M. glyptostroboides* have occasionally been included in reviews which focused on specific compound classes such as flavonoids (Beckmann et al. 1971; Gadek and Quinn 1989; Harborne and Mabry 1982; Hida 1958; Sawada 1958; Takahashi et al. 1960b), carotenoids (Ida 1981a, b) and sugars (Hida et al. 1962). A review reports on sources of shikimic acid including *M. glyptostroboides* (Hattori et al. 1954). Another review, which includes this tree, examines leaf waxes of several deciduous conifers without reporting any chemical constituents (Isoi 1958). The lack of complete, comprehensive literature of natural products from *M. glyptostroboides* has consequently led to cases of double reporting, where previous characterizations from this plant source have been overlooked.

The current review covers six decades of phytochemical investigation of *M. glyptostroboides* (1954–2014). A complete compilation of the considerable number of compounds characterized from *M. glyptostroboides* is presented for the first time (Table 1). Such a compilation may be invaluable for the increasing number of researchers working with natural products from this unique species. The exceptionality of *M. glyptostroboides* necessitates a particular focus on compounds unique to this species including available data regarding their biological activity. Consequently, the current paper also includes the first comprehensive review of studies on various biological activities of extracts and pure compounds from *M. glyptostroboides* as well as current medical applications. Moreover, the potential influence of geographical localization on secondary metabolite production of *M. glyptostroboides* is briefly discussed as this may be particularly relevant in view of the fact that since its rediscovery seven decades ago the tree has been extensively cultivated all over the world in regions where climatic conditions are suitable for this species, mainly covering its original prehistorical habitat.

**Table 1:** Natural products identified from *Metasequoia glyptostroboides* Hu et Cheng

No.	Substances alphabetically according to group	Part of the tree	Methods of identification	References
	*Alcohols*			
1	Ethanol	Leaves	GC–MS	Fujita (1990)
2	Butylcarbinol (pentan-1-ol)	Leaves	GC–MS	Bajpai et al. (2009)
		Leaves	GC–MS	Bajpai and Kang (2011b)
3	*n*-Hexanol	Leaves	GC–MS	Fujita (1990)
		Leaves	N/A	Fujita and Kawai (1991)
4	3-Hexen-1-ol	Shoots	N/A	Fujita et al. (1975)
5	Cis-3-Hexen-1-ol	Leaves	GC–MS	Fujita (1990)
		Leaves	N/A	Fujita and Kawai (1991)
6	Trans-2-Hexen-1-ol	Leaves	GC–MS	Fujita (1990)
7	*n*-Octanol	Leaves	GC–MS	Fujita (1990)
		Leaves	N/A	Fujita and Kawai (1991)
8	l-Octen-3-ol (Amyl vinyl carbinol)	Shoots, branchlet and trunk	N/A	Fujita et al. (1975)
		Leaves	GC–MS	Fujita (1990)
		Leaves	N/A	Fujita and Kawai (1991)
		Leaves	GC–MS	Bajpai and Kang (2011b)
9	7-Octen-2-ol	Leaves	GC–MS	Bajpai et al. (2009)
		Leaves	GC–MS	Bajpai and Kang (2011b)
10	9,12-Tetradecadien-1-ol	Leaves	GC–MS	Bajpai et al. (2009)
		Leaves	GC–MS	Bajpai and Kang (2011b)
11	Ginnol [(+)-*n*-Nonacosanol-(10)]	Leaves	IR, MS, OR	Beckmann and Schuhle (1968)
12	2-Phenyl ethyl alcohol	Leaves	GC–MS	Fujita (1990)
		Leaves	N/A	Fujita and Kawai (1991)
13	4-Methyl-1-(1-methylethyl)-3-cyclohexane-1-ol	Seeds	GC–MS	Mou et al. (2007)
14	3-Cyclohexene-1-ol	Leaves	GC–MS	Bajpai et al. (2009)
		Leaves	GC–MS	Bajpai and Kang (2011b)
15	Sequoyitol	Leaves	PC	Kariyone et al. (1958)
		Leaves	PPC	Takahashi et al. (1960a)
		Heartwood	IR, MP, EA	Sato et al. (1966)
16	Benzyl alcohol	Leaves	GC–MS	Fujita (1990)
		Leaves	N/A	Fujita and Kawai (1991)
		Leaves	GC–MS	Bajpai et al. (2009)
		Leaves	GC–MS	Bajpai and Kang (2011b)
	*Aldehydes*			
17	Benzaldehyde	Leaves	GC–MS	Fujita (1990)
	*Alkanes*			
18	Tetracosane	Leaves	GC–MS	Fujita (1990)
		Fossil leaves	GC–MS	Zhao et al. (2007)
19	Pentacosane	Leaves	GC–MS	Fujita (1990)
		Fossil leaves	GC–MS	Zhao et al. (2007)
20	Cyclobutane	Leaves	GC–MS	Bajpai et al. (2009)
		Leaves	GC–MS	Bajpai and Kang (2011b)
21	Cyclopentane	Leaves	GC–MS	Bajpai et al. (2009)
		Leaves	GC–MS	Bajpai and Kang (2011b)
22	2,3,3-Trimethyl tricycle heptane	Cones	GC–MS	Bajpai et al. (2007a)
		Cones	GC–MS	Bajpai et al. (2007b)
	*Alkynes*			
23	(Z)-3-Heptadecen-5-yne	Cones	GC–MS	Bajpai et al. (2007a)
		Cones	GC–MS	Bajpai et al. (2007b)
24	13-Heptadecyn-1-ol	Cones	GC–MS	Bajpai et al. (2007a)
		Cones	GC–MS	Bajpai et al. (2007b)
25	1-Dodecyn-4-ol	Leaves	GC–MS	Bajpai et al. (2009)
		Leaves	GC–MS	Bajpai and Kang (2011b)
	*Amide*			
26	Valeramide	Leaves	GC–MS	Bajpai et al. (2009)
		Leaves	GC–MS	Bajpai and Kang (2011b)
	*Apocarotenoids*			
27	Icariside B1	Branches and stems	N/A	Zeng et al. (2013)
28	Icariside B1 aglycon	Branches and stems	N/A	Zeng et al. (2013)
29	4′Dihydrophaseic acid	Branches and stems	N/A	Zeng et al. (2013)
30	4′-Dihydrophaseic acid 4′-O-β-D-glucopyranoside	Branches and stems	N/A	Zeng et al. (2013)
	*Dihydrostilbenoids*			
31	6-Carboxydihydroresveratrol 3-*O*-β-glucopyranoside	Leaves	NMR, MS	Nguyen et al. (2014)
	*Esters*			
32	Isopropyl acetate	Cones	GC–MS	Bajpai et al. (2007a)
		Cones	GC–MS	Bajpai et al. (2007b)
33	Methyl 4-methoxybutanoate	Seeds	GC–MS	Mou et al. (2007)
34	Cis-3-Hexenyl acetate	Leaves	GC–MS	Fujita (1990)
		Leaves	N/A	Fujita and Kawai (1991)
35	l-Octen-3-yl acetate	Shoot, branchlet and trunk	N/A	Fujita et al. (1975)
36	Methyl-decanoate	Leaves	GC–MS	Eryin and Rongai (1997)
	*Furans*			
37	Furan	Leaves	GC–MS	Bajpai et al. (2009)
		Leaves	GC–MS	Bajpai and Kang (2011b)
38	5-Ethyl-2(5H)-furanone	Cones	GC–MS	Bajpai et al. (2007a)
		Cones	GC–MS	Bajpai et al. (2007b)
	*Ketones*			
39	2-Butanone	Leaves	GC–MS	Bajpai et al. (2009)
		Leaves	GC–MS	Bajpai and Kang (2011b)
40	3-Pinanone	Seeds	GC–MS	Mou et al. (2007)
41	6,10,14-Trimethyl pentadecan-2-one	Leaves	GC–MS	Fujita (1990)
42	β-Ionone	Leaves	GC–MS	Eryin and Rongai (1997)
43	Acetophenone	Leaves	GC–MS	(Bajpai et al. 2009)
		Leaves	GC–MS	Bajpai and Kang (2011b)
	*Fatty acids and their derivatives*			
44	C_5_H_11_COOH (Hexaenoic acid)	Cones	GC–MS	Bajpai et al. (2007a)
45	C_7_H_15_COOH (Octanoic acid)	Leaves	GC–MS	Bajpai et al. (2009)
		Leaves	GC–MS	Bajpai and Kang (2011b)
46	C_9_H_21_COOH (Capric acid)	Heartwood	GLC	Sato et al. (1966)
47	C_11_H_23_COOH (Lauric acid/Dodecanoic acid)	Heartwood	GLC	Sato et al. (1966)
		Twigs	IR, GC, S	Hayashi et al. (1969)
		Photosynthetic tissue	GLC	Mongrand et al. (2001)
48	C_12_H_25_COOH (Tridecyclic acid/Tridecanoic acid)	Twigs	IR, GC, S	Hayashi et al. (1969)
		Photosynthetic tissue	GLC	Mongrand et al. (2001)
49	C_13_H_27_COOH (Myristic acid/Tetradecanoic acid)	Heartwood	GLC	Sato et al. (1966)
		Twigs	IR, GC, S	Hayashi et al. (1969)
50	C_14_H_29_COOH (Pentadecanoic acid)	Twigs	IR, GC, S	Hayashi et al. (1969)
51	C_14_H_27_COOH	Twigs	IR, GC, S	Hayashi et al. (1969)
52	C_15_H_31_COOH (Palmitic acid/Hexadecanoic acid)	Heartwood	GLC	Sato et al. (1966)
		Twigs	IR, GC, S	Hayashi et al. (1969)
		Leaves	GC–MS	Eryin and Rongai (1997)
		Photosynthetic tissue	GLC	Mongrand et al. (2001)
53	C_15_H_29_COOH	Twigs	IR, GC, S	Hayashi et al. (1969)
		Photosynthetic tissue	GLC	Mongrand et al. (2001)
54	C_15_H_27_COOH	Twigs	IR, GC, S	Hayashi et al. (1969)
55	16:2 Δ7,10	Photosynthetic tissue	GLC	Mongrand et al. (2001)
56	16:3 Δ7,10,13	Photosynthetic tissue	GLC	Mongrand et al. (2001)
57	C_16_H_33_COOH (Margaric acid/Heptadecanoic acid)	Twigs	IR, GC, S	Hayashi et al. (1969)
58	C_17_H_35_COOH (Stearic acid/Octadecanoic acid)	Twigs	IR, GC, S	Hayashi et al. (1969)
		Photosynthetic tissue	GLC	Mongrand et al. (2001)
59	C_17_H_33_COOH (Oleic acid)	Twigs	IR, GC, S	Hayashi et al. (1969)
60	C_17_H_31_COOH (Linoleic acid)	Twigs	IR, GC, S	Hayashi et al. (1969)
61	C_17_H_29_COOH	Twigs	IR, GC, S	Hayashi et al. (1969)
62	18:1 Δ9	Photosynthetic tissue	GLC	Mongrand et al. (2001)
63	18:2 Δ9,12	Photosynthetic tissue	GLC	Mongrand et al. (2001)
64	18:3 Δ9,12,15	Photosynthetic tissue	GLC	Mongrand et al. (2001)
65	C_19_H_39_COOH (Eicosanoic acid/Icosanoic acid)	Twigs	IR, GC, S	Hayashi et al. (1969)
		Photosynthetic tissue	GLC	Mongrand et al. (2001)
66	20:2 Δ5,11	Photosynthetic tissue	GLC	Mongrand et al. (2001)
67	20:2 Δ11,14	Photosynthetic tissue	GLC	Mongrand et al. (2001)
68	20:3 Δ5,11,14	Photosynthetic tissue	GLC	Mongrand et al. (2001)
69	20:4 Δ5,11,14,17	Photosynthetic tissue	GLC	Mongrand et al. (2001)
70	22:0	Photosynthetic tissue	GLC	Mongrand et al. (2001)
71	6,9,12,15-Docosatetraenoic acid	Leaves	GC–MS	Bajpai et al. (2009)
		Leaves	GC–MS	Bajpai and Kang (2011b)
72	Methyl arachidonate	Seeds	GC–MS	Mou et al. (2007)
	*Other carboxylic acids*			
73	2-Hydroxypropanoic acid	Leaves	GC–MS	Bajpai et al. (2009)
		Leaves	GC–MS	Bajpai and Kang (2011b)
74	Shikimic acid	N/A	IR	Hattori et al. (1954)
	*Flavonoids*			
	I. Anthocyanidins			
75	Cyanidin	Leaves and other tissues	PC	Bate-Smith (9^1954^)
		Leaves	PC,S	Hida (1958)
76	Delphinidin	Leaves	PC, S	Hida (1958)
	II. Flavones			
77	Apigenin	Leaves	TLC, UV, MS, NMR	Krauze-Baranowska (2004)
78	Apigenin-7-glucosid (Cosmosiin)	Leaves	TLC, PC	Beckmann and Geiger (1968)
79	Luteolin	Leaves	TLC, UV, MS, NMR	Krauze-Baranowska (2004)
80	Luteolin-7-glucosid	Leaves	TLC, PC	Beckmann and Geiger (1968)
81	Tricetin	Leaves	TLC, PC	Beckmann and Geiger (1968)
82	Tricetin-7-glucosid	Leaves	TLC, PC	Beckmann and Geiger (1968)
83	Tricetin 3′-O-glucoside	Leaves	TLC, UV, MS, NMR	Krauze-Baranowska (2004)
	III. Dihydroflavonols			
84	Aromadendrin-7-O-β-glucopyranoside	Leaves	NMR, MS	Nguyen et al. (2014)
85	Aromadendrin oxide	Leaves	GC–MS	Bajpai et al. (2009)
		Leaves	GC–MS	Bajpai and Kang (2011b)
	IV. Flavonols			
86	Kaempferol	N/A	PC	Takahashi et al. (1960b)
		Leaves	TLC, UV, MS, NMR	Krauze-Baranowska (2004)
87	Kaempferol-3-rhamnosid (Afzelin)	Leaves	TLC, PC	Beckmann and Geiger (1968)
88	Quercetin	N/A	PC	Takahashi et al. (1960b)
		Leaves	UV	Katou and Homma (1996)
		Leaves	TLC, UV, MS, NMR	Krauze-Baranowska (2004)
89	Quercetin-3-rhamnosid (Quercitrin)	Leaves	PPC	Takahashi et al. (1960a)
		Leaves	TLC, PC	Beckmann and Geiger (1968)
		Leaves	UV	Katou and Homma (1996)
		Leaves	MP, UV, MS, NMR	Duan et al. (2009)
90	Quercetin 3-glucoside (isoquercetin, isoquercitrin)	Leaves	MP, MS, NMR, UV	Duan et al. (2009)
91	Quercetin-3-*O*-α-rhamnopyranoside-7-O-β-glucopyranoside	Leaves	NMR, MS	Nguyen et al. (2014)
92	Isorhamnetin	N/A	PC	Takahashi et al. (1960b)
93	Myricetin	N/A	PC	Takahashi et al. (1960b)
		Leaves	TLC, PC	Beckmann and Geiger (1968)
94	Myricetin-3-rhamnosid (Myricitrin)	Leaves	MP, MS, NMR, UV	Duan et al. (2009)
	V. Flavanols			
95	Catechin	Heartwood	IR, TLC, MP, EA	Sato et al. (1966)
		Branches and stems	N/A	Zeng et al. (2013)
		Bark	NMR, MS	Chen et al. (2014)
96	Epicatechin	Heartwood	IR, TLC, MP, EA	Sato et al. (1966)
		Branches and stems	N/A	Zeng et al. (2013)
		Bark	NMR, MS	Chen et al. (2014)
97	Gallocatechin	Branches and stems	N/A	Zeng et al. (2013)
		Leaves	NMR, MS	Nguyen et al. (2014)
		Bark	NMR, MS	Chen et al. (2014)
98	Epi-Gallocatechin	Branches and stems	N/A	Zeng et al. (2013)
		Bark	NMR, MS	Chen et al. (2014)
	*Dimeric flavonoids*			
	I. Biflavones and Bi(flavone + flavanone)			
99	Amentoflavone	Leaves	TLC, UV	Gadek and Quinn (1989)
		Leaves	MP, MS, NMR, UV	Duan et al. (2009)
100	7-Monomethyl Amentoflavone (Sequoiaflavone)	Leaves	TLC, UV	Gadek and Quinn (1989)
101	4′-Monomethyl Amentoflavone (Podocarpus flavone A)	Leaves	TLC, UV	Gadek and Quinn (1989)
102	7, 4′″-Dimethyl Amentoflavone (Podocarpus flavone B)	Leaves	TLC, UV	Gadek and Quinn (1989)
103	4′,4″-Dimethyl Amentoflavone (Isoginkgetin)	Leaves	TLC, UV	Gadek and Quinn (1989)
104	7, 4′,4″-Trimethyl Amentoflavone (Sciadopitysin)	Leaves	TLC, UV	Gadek and Quinn (1989)
105	2,3-Dihydro dimethyl Amentoflavone	Leaves	TLC, UV	Gadek and Quinn (1989)
106	2,3-Dihydroamentoflavone-7″,4′″-dimethylether	Leaves	NMR, MP	Beckmann et al. (1971)
107	Amentoflavone-7″,4′″-dimethyl ether	Leaves	NMR, MP	Beckmann et al. (1971)
108	Bilobetin	Leaves	TLC, UV, MS, NMR	Krauze-Baranowska (2004)
109	Ginkgetin	Leaves	TLC, UV, MS, NMR	Krauze-Baranowska (2004)
110	Hinokiflavone	Leaves	N/A	Sawada (1958)
		Leaves	PC	Kariyone et al. (1958)
		Leaves	NMR, MP	Beckmann et al. (1971)
		Leaves	TLC, UV	Gadek and Quinn (1989)
111	Isocryptomerin	Leaves	NMR, MP	Beckmann et al. (1971)
112	Isoginkgetin	Leaves	MP, MS, NMR, UV	Duan et al. (2009)
113	Robustaflavone	Leaves	TLC, UV	Gadek and Quinn (1989)
114	Sciadopitysin	Leaves	MP, MS, NMR, UV	Duan et al. (2009)
115	Sotetsuflavone	Leaves	NMR, MP	Beckmann et al. (1971)
116	2,3-Dihydrohinokiflavone	Leaves	NMR, MP	Beckmann et al. (1971)
		Leaves	TLC, UV	Gadek and Quinn (1989)
117	2,3-Dihydroisoginkgetin	Leaves	TLC, UV, MS, NMR	Krauze-Baranowska (2004)
118	2,3-Dihydrosciadopitysin	Leaves	MP, MS, NMR, UV	Duan et al. (2009)
	II. Biflavanols			
119	Catechin (4 → 8) Catechin	Bark	NMR, MS	Chen et al. (2014)
120	Gallocatechin (4 → 8) Gallocatechin	Bark	NMR, MS	Chen et al. (2014)
121	Gallocatechin (4 → 8) Epigallocatechin	Bark	NMR, MS	Chen et al. (2014)
122	Gallocatechin (4 → 8) Catechin	Bark	NMR, MS	Chen et al. (2014)
123	Catechin (4 → 8) Gallocatechin	Bark	NMR, MS	Chen et al. (2014)
124	Gallocatechin (4 → 8) Epicatechin	Bark	NMR, MS	Chen et al. (2014)
	*Hydrocarbons*			
125	1-Methyl-4-(1-methylethyl)-benzene	Seeds	GC–MS	Mou et al. (2007)
126	1,2,3,4,4a,9,10,10α -Octahydro-1-phenanthrene	Seeds	GC–MS	Mou et al. (2007)
127	1,6,10-Dodecatriene	Leaves	GC–MS	Bajpai et al. (2009)
128	Ethylene (Ethene)	Stems	GC-FID	Du et al. (2004)
		Leaves	GC–MS	Bajpai and Kang (2011b)
129	Tricyclene	Leaves	GC–MS	Eryin and Rongai (1997)
		Cones	GC–MS	Bajpai et al. (2007a)
	*Aromatic hydrocarbons*			
130	Perylene	Cones	GC–MS	Bajpai et al. (2007a)
	*Lignans*			
131	Arctigenin	Branches and stems	N/A	Zeng et al. (2013)
132	+(−)Lariciresinol	Branches and stems	N/A	Zeng et al. (2013)
133	Matairesinol	Branches and stems	N/A	Zeng et al. (2013)
134	(−)-Meridinol	Branches and stems	N/A	Zeng et al. (2013)
135	Pinopalustrin	Branches and stems	N/A	Zeng et al. (2013)
136	Pinoresinol	Branches and stems	N/A	Zeng et al. (2013)
137	Thujastandin	Branches and stems	N/A	Zeng et al. (2013)
138	1-(4-hydroxy-3-methoxyphenyl)-2-[4-(3-hydroxypropyl)-2-methoxyphenoxy]-propane-1,3-diol	Branches and stems	N/A	Zeng et al. (2013)
139	2-[2-hydroxy-4-(3-hydroxypropyl)phenoxy]-1-(4-hydroxy-3-methoxyphenyl)-1,3-propanediol	Branches and stems	N/A	Zeng et al. (2013)
140	(7S,8S)-3-methoxy-3′,7-epoxy-4′,8-oxyneoligna-4,9,9′-triol	Branches and stems	N/A	Zeng et al. (2013)
	*Norlignans*			
141	Agatharesinol	Heartwood	UV, IR, OR, NMR	Enoki et al. (1977a)
		Branches and stems	N/A	Zeng et al. (2013)
142	Athrotaxin	Heartwood	UV, IR, OR, NMR	Enoki et al. (1977a)
		Heartwood	N/A	Nagasaki et al. (2004)
143	Hydroxyathrotaxin	Heartwood	MP, OR, IR, UV, MS, NMR	Enoki et al. (1977b)
144	(−)-Evofolin	Branches and stems	N/A	Zeng et al. (2013)
145	Ficusal	Branches and stems	N/A	Zeng et al. (2013)
146	Metasequirin A	Heartwood	UV, IR, MS, NMR	Enoki et al. (1977a)
		Branches and stems	N/A	Zeng et al. (2013)
147	Hydroxymetasequirin A	Heartwood	MS, IR, UV, NMR	Enoki et al. (1977b)
		Branches and stems	N/A	Zeng et al. (2013)
148	Metasequirin B	Heartwood	MP, MS, IR, UV, NMR	Enoki et al. (1977b)
149	Metasequirin C	Heartwood	N/A	Nagasaki et al. (2004)
150	Metasequirin D	Stems and leaves	IR, MS, NMR, OR, UV	Dong et al. (2011)
151	Metasequirin E	Stems and leaves	IR, MS, NMR, UV	Dong et al. (2011)
152	Metasequirin F	Stems and leaves	IR, MS, NMR, UV	Dong et al. (2011)
153	Metasequirin G	Branches and stems	NMR, MS	Zeng et al. (2012)
154	Metasequirin H	Branches and stems	NMR, MS	Zeng et al. (2012)
155	Metasequirin I	Branches and stems	NMR, MS	Zeng et al. (2012)
156	Sequirin C	Branches and stems	N/A	Zeng et al. (2013)
157	Sequosempervirin B	Branches and stems	N/A	Zeng et al. (2013)
158	Sequosempervirin F	Branches and stems	N/A	Zeng et al. (2013)
159	Threo-2,3-bis-(4-hydroxy-3-methoxyphenyl)-3-raethoxypropanol	Branches and stems	N/A	Zeng et al. (2013)
160	7′R,8′S-Threoguaiacylglycerol 8′-vanillic acid ether	Branches and stems	N/A	Zeng et al. (2013)
161	7′S,8′R-Threoguaiacylglycerol 8’-vanillic acid ether	Branches and stems	N/A	Zeng et al. (2013)
	*Quinic acid derivatives*			
162	3-*O*-(*E*)-Coumaroylquinic acid	Leaves	NMR, MS	Nguyen et al. (2014)
163	3-*O*-(*Z*)-Coumaroylquinic acid	Leaves	NMR, MS	Nguyen et al. (2014)
164	3-*O*-(*E*)-Coumaroylquinic acid methyl ester	Leaves	NMR, MS	Nguyen et al. (2014)
165	3-*O*-(*Z*)-Coumaroylquinic acid methyl ester	Leaves	NMR, MS	Nguyen et al. (2014)
	*Terpenoids*			
	I. Monoterpenoids			
166	Borneol	Leaves	GC–MS	Eryin and Rongai (1997)
		Leaves	GC–MS	Bajpai et al. (2009)
		Leaves	GC–MS	Bajpai and Kang (2011b)
167	Bornneol formate	Cones	GC–MS	Bajpai et al. (2007a)
		Cones	GC–MS	Bajpai et al. (2007b)
168	Bornylene	Cones	GC–MS	Bajpai et al. (2007a)
		Cones	GC–MS	Bajpai et al. (2007b)
169	Endo bornyl acetate	Cones	GC–MS	Bajpai et al. (2007a)
		Cones	GC–MS	Bajpai et al. (2007b)
170	Exo bornyl acetate	Cones	GC–MS	Bajpai et al. (2007a)
		Cones	GC–MS	Bajpai et al. (2007b)
171	Isobornyl acetate	Leaves	GC–MS	Bajpai et al. (2009)
		Leaves	GC–MS	Bajpai and Kang (2011b)
172	Camphene	Shoot, branchlet and trunk	N/A	Fujita et al. (1975)
		Leaves	GC–MS	Fujita (1990)
		Leaves	N/A	Fujita and Kawai (1991)
		Leaves	GC–MS	Eryin and Rongai (1997)
		Seeds	GC–MS	Mou et al. (2007)
173	Camphene hydrate	Leaves	GC–MS	Fujita (1990)
		Leaves	GC–MS	Eryin and Rongai (1997)
174	α-Campholenone aldehyde	Leaves	GC–MS	Fujita (1990)
175	α-Campholene aldehyde	Leaves	GC–MS	Eryin and Rongai (1997)
176	Camphor	Shoot, branchlet and trunk	N/A	Fujita et al. (1975)
177	Cis-Carane	Cones	GC–MS	Bajpai et al. (2007a)
		Cones	GC–MS	Bajpai et al. (2007b)
178	δ-3-Carene	Shoot, branchlet and trunk	N/A	Fujita et al. (1975)
		Leaves	GC–MS	Eryin and Rongai (1997)
		Cones	GC–MS	Bajpai et al. (2007a)
		Cones	GC–MS	Bajpai et al. (2007b)
		Seeds	GC–MS	Mou et al. (2007)
179	3-Caren-4-ol	Leaves	GC–MS	Bajpai et al. (2009)
		Leaves	GC–MS	Bajpai and Kang (2011b)
180	Carnosol	Cones	GC–MS	Bajpai et al. (2007a)
		Cones	GC–MS	Bajpai et al. (2007b)
181	Trans-carveol	Leaves	GC–MS	Fujita (1990)
		Leaves	N/A	Fujita and Kawai (1991)
182	Carvone	Shoot, branchlet and trunk	N/A	Fujita et al. (1975)
183	*p*-Cymene	Shoot, branchlet and trunk	N/A	Fujita et al. (1975)
		Leaves	GC–MS	Fujita (1990)
		Leaves	N/A	Fujita and Kawai (1991)
		Leaves	GC–MS	Eryin and Rongai (1997)
184	*p*-Cymene-8-ol	Leaves	GC–MS	Fujita (1990)
		Leaves	N/A	Fujita and Kawai (1991)
185	Dihydrocarvyl acetate	Cones	GC–MS	Bajpai et al. (2007a)
		Cones	GC–MS	Bajpai et al. (2007b)
186	Cyclofenchene	Cones	GC–MS	Bajpai et al. (2007a)
		Cones	GC–MS	Bajpai et al. (2007b)
187	1,8-Cineole	Shoot, branchlet and trunk	N/A	Fujita et al. (1975)
188	Citronellyl acetate	Cones	GC–MS	Bajpai et al. (2007a)
		Cones	GC–MS	Bajpai et al. (2007b)
189	α-Fenchene	Seeds	GC–MS	Mou et al. (2007)
190	Fenchol	Leaves	GC–MS	Bajpai et al. (2009)
		Leaves	GC–MS	Bajpai and Kang (2011b)
191	Fenchone	Shoot, branchlet and trunk	N/A	Fujita et al. (1975)
192	α-Fenchyl alcohol	Leaves	GC–MS	Fujita (1990)
193	Geraniol	Leaves	GC–MS	Fujita (1990)
		Leaves	N/A	Fujita and Kawai (1991)
194	Geranyl acetate	Leaves	N/A	Fujita and Kawai (1991)
		Cones	GC–MS	Bajpai et al. (2007a)
		Cones	GC–MS	Bajpai et al. (2007b)
195	Geranyl bromide	Leaves	GC–MS	Bajpai et al. (2009)
		Leaves	GC–MS	Bajpai and Kang (2011b)
196	Homomyrtenol	Cones	GC–MS	Bajpai et al. (2007a)
		Cones	GC–MS	Bajpai et al. (2007b)
197	Limonene	Shoot, branchlet and trunk	N/A	Fujita et al. (1975)
		Leaves	GC–MS	Fujita (1990)
		Leaves	N/A	Fujita and Kawai (1991)
		Leaves	GC–MS	Eryin and Rongai (1997)
		Cones	GC–MS	Bajpai et al. (2007a)
		Cones	GC–MS	Bajpai et al. (2007b)
		Seeds	GC–MS	Mou et al. (2007)
		Leaves	GC–MS	Bajpai et al. (2009)
		Leaves	GC–MS	Bajpai and Kang (2011b)
198	*cis*-Limonene oxide	Seeds	GC–MS	Mou et al. (2007)
199	Linalool	Shoot, branchlet and trunk	N/A	Fujita et al. (1975)
		Leaves	GC–MS	Fujita (1990)
		Leaves	N/A	Fujita and Kawai (1991)
200	Linalool oxide	Leaves	GC–MS	Bajpai et al. (2009)
		Leaves	GC–MS	Bajpai and Kang (2011b)
201	Trans-Linalool oxide	Leaves	GC–MS	Fujita (1990)
		Leaves	N/A	Fujita and Kawai (1991)
202	Cis-Linalool oxide	Leaves	GC–MS	(Fujita 1990)
		Leaves	N/A	(Fujita and Kawai 1991)
203	Linalyl acetate	Shoot, branchlet and trunk	N/A	Fujita et al. (1975)
		Cones	GC–MS	Bajpai et al. (2007a)
		Cones	GC–MS	Bajpai et al. (2007b)
204	Linaloyl propionate	Cones	GC–MS	Bajpai et al. (2007a)
		Cones	GC–MS	Bajpai et al. (2007b)
205	Methylol pinene (Nopol)	Cones	GC–MS	Bajpai et al. (2007a)
206	Myrcene	Leaves	GC–MS	Eryin and Rongai (1997)
207	β-Myrcene	Leaves	GC–MS	Fujita (1990)
		Leaves	N/A	Fujita and Kawai (1991)
		Cones	GC–MS	Bajpai et al. (2007a)
		Cones	GC–MS	Bajpai et al. (2007b)
		Seeds	GC–MS	Mou et al. (2007)
		Leaves	GC–MS	Bajpai et al. (2009)
		Leaves	GC–MS	Bajpai and Kang (2011b)
208	Myrtenol	Leaves	GC–MS	Fujita (1990)
		Leaves	N/A	Fujita and Kawai (1991)
		Cones	GC–MS	Bajpai et al. (2007a)
		Cones	GC–MS	Bajpai et al. (2007b)
		Leaves	GC–MS	Bajpai et al. (2009)
		Leaves	GC–MS	Bajpai and Kang (2011b)
209	Nerol	Leaves	GC–MS	Fujita (1990)
		Leaves	N/A	Fujita and Kawai (1991)
210	Nopyl acetate	Cones	GC–MS	Bajpai et al. (2007a)
		Cones	GC–MS	Bajpai et al. (2007b)
211	Ocimene	Leaves	GC–MS	Bajpai et al. (2009)
		Leaves	GC–MS	Bajpai and Kang (2011b)
212	Perilla-aldehyde (tentative identification)	Shoot, branchlet and trunk	N/A	Fujita et al. (1975)
213	α-Phellandrene^a^	Leaves	GC–MS	Fujita (1990)
		Leaves	N/A	Fujita and Kawai (1991)
214	1-Phellandrene^a^	Seeds	GC–MS	Mou et al. (2007)
215	β-Phellandrene	Leaves	GC–MS	Fujita (1990)
		Leaves	N/A	Fujita and Kawai (1991)
		Leaves	GC–MS	Eryin and Rongai (1997)
		Seeds	GC–MS	Mou et al. (2007)
216	α-Pinene^b^	Heartwood	GLC	Sato et al. (1966)
		Leaves	GC–MS	Fujita (1990)
		Leaves	N/A	Fujita and Kawai (1991)
		Leaves	GC–MS	Eryin and Rongai (1997)
		Cones	GC–MS	Bajpai et al. (2007a)
		Cones	GC–MS	Bajpai et al. (2007b)
		Seeds	GC–MS	Mou et al. (2007)
		Leaves	GC–MS	Bajpai et al. (2009)
		Leaves	GC–MS	Bajpai and Kang (2011b)
217	l-α-Pinene^b^	Shoot, branchlet and trunk	N/A	Fujita et al. (1975)
218	β-Pinene^c^	Shoot, branchlet and trunk	N/A	Fujita et al. (1975)
		Leaves	GC–MS	Fujita (1990)
		Leaves	N/A	Fujita and Kawai (1991)
		Leaves	GC–MS	Eryin and Rongai (1997)
		Seeds	GC–MS	Mou et al. (2007)
219	1-β-Pinene^c^	Cones	GC–MS	Bajpai et al. (2007a)
		Cones	GC–MS	Bajpai et al. (2007b)
220	2-β-Pinene	Cones	GC–MS	Bajpai et al. (2007a)
		Cones	GC–MS	Bajpai et al. (2007b)
221	2-Pinen-4-ol	Leaves	GC–MS	Bajpai et al. (2009)
		Leaves	GC–MS	Bajpai and Kang (2011b)
222	Trans-Pinocarcved	Leaves	GC–MS	Eryin and Rongai (1997)
223	Sabinene	Leaves	GC–MS	Fujita (1990)
		Leaves	N/A	Fujita and Kawai (1991)
		Leaves	GC–MS	Eryin and Rongai (1997)
		Cones	GC–MS	Bajpai et al. (2007a)
		Cones	GC–MS	Bajpai et al. (2007b)
		Seeds	GC–MS	Mou et al. (2007)
224	Cis-Sabinenehydrate	Cones	GC–MS	Bajpai et al. (2007a)
		Cones	GC–MS	Bajpai et al. (2007b)
225	α-Terpineol	Shoot, branchlet and trunk	N/A	Fujita et al. (1975)
		Leaves	GC–MS	Fujita (1990)
		Leaves	N/A	Fujita and Kawai (1991)
		Leaves	GC–MS	Eryin and Rongai (1997)
		Cones	GC–MS	Bajpai et al. (2007a)
		Cones	GC–MS	Bajpai et al. (2007b)
		Leaves	GC–MS	Bajpai et al. (2009)
		Leaves	GC–MS	Bajpai and Kang (2011b)
226	δ-Terpineol	Leaves	GC–MS	Fujita (1990)
		Leaves	N/A	Fujita and Kawai (1991)
227	α-Terpinene	Leaves	GC–MS	Fujita (1990)
		Cones	GC–MS	Bajpai et al. (2007a)
		Cones	GC–MS	Bajpai et al. (2007b)
228	δ-Terpinene	Seeds	GC–MS	Mou et al. (2007)
229	γ-Terpinene	Leaves	GC–MS	Fujita (1990)
		Leaves	N/A	Fujita and Kawai (1991)
		Cones	GC–MS	Bajpai et al. (2007b)
230	Terpinen-4-ol^d^	Shoot, branchlet and trunk	N/A	Fujita et al. (1975)
		Leaves	GC–MS	Fujita (1990)
		Leaves	N/A	Fujita and Kawai (1991)
		Leaves	GC–MS	Eryin and Rongai (1997)
		Cones	GC–MS	Bajpai et al. (2007b)
231	Terpinolene	Leaves	GC–MS	Fujita (1990)
		Leaves	N/A	Fujita and Kawai (1991)
232	α-Terpinolene	Leaves	GC–MS	Eryin and Rongai (1997)
		Seeds	GC–MS	Mou et al. (2007)
233	γ-Terpinolene	Leaves	GC–MS	Eryin and Rongai (1997)
234	Terpitineol-4^d^	Cones	GC–MS	Bajpai et al. (2007b)
235	α-Terpinyl acetate	Shoot, branchlet and trunk	N/A	Fujita et al. (1975)
236	α-Thujene	Leaves	GC–MS	Fujita (1990)
		Cones	GC–MS	Bajpai et al. (2007a)
		Cones	GC–MS	Bajpai et al. (2007b)
237	Thymol	Leaves	GC–MS	Bajpai et al. (2009)
		Leaves	GC–MS	Bajpai and Kang (2011b)
238	Tricyclene	Cones	GC–MS	Bajpai et al. (2007b)
		Seeds	GC–MS	Mou et al. (2007)
239	Verbenol	Leaves	GC–MS	Bajpai et al. (2009)
		Leaves	GC–MS	Bajpai and Kang (2011b)
	II. Sesquiterpenoids			
240	(−)-Acora-2,4(14),8-trien-15-oic acid	Stems, leaves	IR, MS, NMR, UV	Dong et al. (2011)
241	Bergamotene	Leaves	GC–MS	Bajpai and Kang (2011b)
242	α-Bisabolol	Leaves	GC–MS	Bajpai and Kang (2011b)
243	α-Bisabolene epoxide	Leaves	GC–MS	Bajpai and Kang (2011b)
244	β-Bisabolene	Leaves	GC–MS	Fujita (1990)
		Cones	GC–MS	Bajpai et al. (2007b)
245	β-Bourbonene	Leaves	GC–MS	Fujita (1990)
		Leaves	N/A	Fujita and Kawai (1991)
		Leaves	GC–MS	Eryin and Rongai (1997)
246	α-Cadinol (C_15_H_26_O_1_)	Twigs	IR,GC, standard	Hayashi et al. (1969)
		Shoot, branchlet and trunk	N/A	Fujita et al. (1975)
		Leaves	GC–MS	Fujita (1990)
		Leaves	N/A	Fujita and Kawai (1991)
247	δ-Cadinol	Leaves	GC–MS	Fujita (1990)
248	δ-Cadinene	Leaves	GC–MS	Fujita (1990)
		Leaves	N/A	Fujita and Kawai (1991)
249	Calamenene	Shoot, branchlet and trunk	N/A	Fujita et al. (1975)
250	Calacorene	Leaves	GC–MS	Fujita (1990)
251	α-Calacorene	Shoot, branchlet and trunk	N/A	Fujita et al. (1975)
252	Caryophylla-1(12),8(15)-dien-9α-ol	Leaves	GC–MS	Fujita (1990)
		Leaves	N/A	Fujita and Kawai (1991)
253	Caryophylla-1(12),8(15)-dien-9β-ol	Leaves	GC–MS	Fujita (1990)
		Leaves	N/A	Fujita and Kawai (1991)
254	Caryophylla-1(12),7-dien-9α-ol	Leaves	GC–MS	Fujita (1990)
		Leaves	N/A	Fujita and Kawai (1991)
255	Caryophylla-1(12),7-dien-9β-ol	Leaves	GC–MS	Fujita (1990)
		Leaves	N/A	Fujita and Kawai (1991)
256	Caryophylla-1(12),7-dien-9-one	Leaves	N/A	Fujita and Kawai (1991)
257	Caryophylla-1(12),8(15)-dien-9-one	Leaves	N/A	Fujita and Kawai (1991)
258	Caryophyllene	Shoot, branchlet and trunk	N/A	Fujita et al. (1975)
259	β-Caryophyllene	Leaves	GC–MS	Fujita (1990)
		Leaves	N/A	Fujita and Kawai (1991)
		Cones	GC–MS	Bajpai et al. (2007a)
		Cones	GC–MS	Bajpai et al. (2007b)
260	γ-Caryophyllene	Cones	GC–MS	Bajpai et al. (2007a)
		Cones	GC–MS	Bajpai et al. (2007b)
261	Caryophyllene oxide	Leaves	GC–MS	Fujita (1990)
		Leaves	N/A	Fujita and Kawai (1991)
		Leaves	GC–MS	Eryin and Rongai (1997)
		Cones	GC–MS	Bajpai et al. (2007a)
		Cones	GC–MS	Bajpai et al. (2007b)
		Leaves	GC–MS	Bajpai et al. (2009)
		Leaves	GC–MS	Bajpai and Kang (2011b)
262	9,3 H-Caryophyllene	Leaves	N/A	Fujita and Kawai (1991)
263	Isocaryophyllene	Leaves	N/A	Fujita and Kawai (1991)
264	Trans-Caryophyllene	Leaves	GC–MS	(Eryin and Rongai 1997)
		Seeds	GC–MS	(Mou et al. 2007)
265	α-Chamigrene	Cones	GC–MS	Bajpai et al. (2007b)
266	β-Cubebene	Leaves	GC–MS	Eryin and Rongai (1997)
267	(R)-Cuparene	Cones	GC–MS	Bajpai et al. (2007b)
268	α-Elemene	Shoot, branchlet and trunk	N/A	Fujita et al. (1975)
269	β-Elemene	Shoot, branchlet and trunk	N/A	Fujita et al. (1975)
		Leaves	GC–MS	Fujita (1990)
270	β-Famesene	Leaves	GC–MS	Bajpai et al. (2009)
271	α-Farnesene	Leaves	GC–MS	Bajpai and Kang (2011b)
272	Trans-β-Farnesene	Leaves	GC–MS	Fujita (1990)
		Leaves	N/A	Fujita and Kawai (1991)
273	Cis-Farnesol	Cones Cones	GC–MS GC–MS	Bajpai et al. (2007a, b)
274	Hexahydrofarnesylacetone	Leaves	GC–MS	Eryin and Rongai (1997)
275	Humuladiene I: C_15_H_24_O	Leaves	GC–MS	Fujita (1990)
276	Humuladiene II: C_15_H_24_O	Leaves	GC–MS	Fujita (1990)
277	Humuladienone I	Leaves	N/A	Fujita and Kawai (1991)
278	Humuladienone II	Leaves	N/A	Fujita and Kawai (1991)
279	α-Humulene	Shoot, branchlet and trunk	N/A	Fujita et al. (1975)
		Leaves	GC–MS	Fujita (1990)
		Leaves	N/A	Fujita and Kawai (1991)
		Cones	GC–MS	Bajpai et al. (2007b)
280	Humulene epoxide I	Leaves	N/A	Fujita and Kawai (1991)
281	Humulene epoxide II	Leaves	N/A	Fujita and Kawai (1991)
282	Humulenol II (tentative identification)	Leaves	N/A	Fujita and Kawai (1991)
283	Hurnulene	Leaves	GC–MS	Eryin and Rongai (1997)
284	Longipinenepoxide	Cones	GC–MS	Bajpai et al. (2007a)
		Cones	GC–MS	Bajpai et al. (2007b)
285	T-Muurolol	Leaves	N/A	Fujita and Kawai (1991)
286	Nerolidol	Leaves	GC–MS	Fujita (1990)
		Leaves	N/A	Fujita and Kawai (1991)
287	β-Selinene	Cones	GC–MS	Bajpai et al. (2007a)
		Cones	GC–MS	Bajpai et al. (2007b)
288	Solanone	Cones	GC–MS	Bajpai et al. (2007a)
		Cones	GC–MS	Bajpai et al. (2007b)
289	Spathulenol	Leaves	GC–MS	Fujita (1990)
290	γ-Terpinine	Cones	GC–MS	Bajpai et al. (2007b)
291	Veridiflorol	Leaves	GC–MS	Bajpai and Kang (2011b)
292	α-Ylangene	Shoot, branchlet and trunk	N/A	Fujita et al. (1975)
293	C_15_H_24_O	Leaves	GC–MS	Fujita (1990)
294	C_15_H_22_O	Leaves	GC–MS	Fujita (1990)
	III. Diterpenoids and their derivatives			
295	Ferruginol	Cones	GC–MS	Bajpai et al. (2007a)
		Cones	GC–MS	Bajpai et al. (2007b)
296	3-Acetoxylabda-8(20),13-dien-15-oic acid	Brown autumn leaves	^13^C NMR	Braun and Breitenbach (1977)
297	3β-Acetoxy-8 (17),13E-labdadien-15-oic acid	Leaves	MP, MS, NMR,	Duan et al. (2009)
298	12α-Hydroxy-8,15-isopimaradien-18-oic acid	Stems, leaves	IR, MS, NMR, UV,	Dong et al. (2011)
299	Metaseglyptorin A	Stems and leaves	IR, MS, MP, NMR, UV	Dong et al. (2011)
300	Metasequoic acid A	Twig (branch)	NMR	Sakan et al. (1988)
301	Metasequoic acid B	Twig (branch)	NMR	Sakan et al. (1988)
302	Metasequoic acid C	Stems, leaves	IR, MS, NMR, UV	Dong et al. (2011)
303	Phytol	Leaves	GC–MS	(Fujita (1990)
		Leaves	N/A	Fujita and Kawai (1991)
304	Sugiol	Cones	GC–MS	Bajpai et al. (2007b)
		Cones	MP, NMR	Bajpai and Kang (2011a)
		Cones	MP, NMR	Bajpai et al. (2014a)
305	Taxaquinone	Cones	IR, MP, NMR, OR, TLC, UV	Bajpai and Kang (2014)
306	Taxodone	Cones	MP, NMR	Bajpai and Kang (2010a)
307	Totarol	Cones	GC–MS	Bajpai et al. (2007a)
		Cones	GC–MS	Bajpai et al. (2007b)
308	Totarol acetate	Cones	GC–MS	Bajpai et al. (2007a)
		Cones	GC–MS	Bajpai et al. (2007b)
309	2-Pentenoic acid, 5-(decahydro-6-hydroxy-5,5,8a-trimethyl-1-naphthalenyl)-3-methyl-, [1S-(1α,4aβ,6α,8aα)]- (9CI)	N/A	NMR, IR, MS	Asahi and Sakan (1984)
310	2-Pentenoic acid, 5-[6-(acetyloxy)decahydro-5,5,8a-trimethyl-1-naphthalenyl]-3-methyl-, [1S-(1α,4aβ,6α,8aα)]- (9CI)	N/A	NMR, IR, MS	Asahi and Sakan (1984)
311	2-Pentenoic acid, 5-(decahydro-5,5,8a-trimethyl-1-naphthalenyl)-3-methyl-, [1R-(1α,4aβ,8aα)]- (9CI)	N.A.	NMR, IR, MS	Asahi and Sakan (1984)
	IV. Triterpenoids			
312	Metaseglyptorin A	Leaves	NMR, MS, IR	Dong et al. (2011)
	V. Tetraterpenoids (Carotenoids)			
313	Adonirubin	Leaves	TLC	Czeczuga (1987)
314	Antheraxanthin	Leaves	TLC	Czeczuga (1987)
315	Apo-12′-violaxanthal	Leaves	TLC	Czeczuga (1987)
316	Astaxanthin	Leaves	TLC	Czeczuga (1987)
317	Auroxanthin	Leaves	TLC	Czeczuga (1987)
318	Canthaxanthin	Leaves	TLC	Czeczuga (1987)
319	α-Carotene	Leaves	UV	Hida and Ida (1961)
		Leaves	UV	Ida (1981b)
		Leaves	UV	Ida (1981a)
		Leaves	TLC	Czeczuga (1987)
320	β-Carotene	Leaves	UV	Ida (1981b)
		Leaves	UV	Ida (1981a)
		Leaves	TLC	Czeczuga (1987)
321	γ-Carotene	Leaves	TLC	Czeczuga (1987)
322	α-Cryptoxanthin	Leaves	TLC	Czeczuga (1987)
323	β-Cryptoxanthin	Leaves	TLC	Czeczuga (1987)
324	Lycopene	Leaves	TLC	Czeczuga (1987)
325	Lutein	Leaves	UV	Hida and Ida (1961)
		Leaves	UV	Ida (1981b)
		Leaves	UV	Ida (1981a)
		Leaves	TLC	Czeczuga (1987)
326	Lutein epoxide	Leaves	TLC	Czeczuga (1987)
327	Luteoxanthin	Leaves	TLC	Czeczuga (1987)
328	Mutatochrome	Leaves	TLC	Czeczuga (1987)
329	Mutatoxanthin	Leaves	TLC	Czeczuga (1987)
330	Neoxanthin	Leaves	TLC	Czeczuga (1987)
331	Rhodoxanthin	Leaves	TLC	Czeczuga (1987)
332	Violaxanthin	Leaves	UV	Hida and Ida (1961)
		Leaves	UV	Ida (1981b)
		Leaves	UV	Ida (1981a)
		Leaves	TLC	Czeczuga (1987)
333	Zeaxanthin	Leaves	TLC	Czeczuga (1987)
	*Phenolic compounds*			
334	p-Cresol	Leaves	GC–MS	Fujita (1990)
		Leaves	N/A	Fujita and Kawai (1991)
335	Metaseol	Root bark	IR, MP, MS, NMR, UV	Nakatani et al. (1991)
336	Phenol	Leaves	GC–MS	Fujita (1990)
		Leaves	N/A	Fujita and Kawai (1991)
		Leaves	GC–MS	Bajpai and Kang (2011b)
337	Protocatechuic acid	Heartwood	IR, TLC, MP, EA	Sato et al. (1966)
338	Protocatechuic aldehyde	Heartwood	IR, TLC, MP, EA	Sato et al. (1966)
	*Phenylpropans*			
339	7-(3-ethoxy-5-methoxyphenyl)propane-7,8,9-triol (1-(3-ethoxy-5-methoxyphenyl)propane-1,2,3-triol)	Branches and stems	NMR, MS	Zeng et al. (2012)
340	7-(3-hydroxy-5-methoxyphenyl)propane-7,8,9-triol (1-(3-hydroxy-5-methoxyphenyl)propane-1,2,3-triol)	Branches and stems	NMR, MS	Zeng et al. (2012)
	*Phenylpropens*			
341	Chavicol	Leaves	GC–MS	Bajpai et al. (2009)
		Leaves	GC–MS	Bajpai and Kang (2011b)
342	Eugenol	Leaves	GC–MS	Bajpai et al. (2009)
		Leaves	GC–MS	Bajpai and Kang (2011b)
343	Guaiacol	Leaves	GC–MS	Bajpai et al. (2009)
		Leaves	GC–MS	Bajpai and Kang (2011b)
	*N-heterocycles*			
344	2,3-Benzopyrrole	Leaves	GC–MS	Bajpai et al. (2009)
		Leaves	GC–MS	Bajpai and Kang (2011b)
345	2-Cyanoaziridine	Cones	GC–MS	Bajpai et al. (2007a)
		Cones	GC–MS	Bajpai et al. (2007b)
346	2,3-Dimethyl 1,3 isopropylpyrazine	Leaves	GC–MS	Bajpai et al. (2009)
		Leaves	GC–MS	(Bajpai and Kang 2011b)
347	Imidazole	Leaves	GC–MS	Bajpai et al. (2009)
348	Indole-3-acetic acid	Stems	GC–MS	Du et al. (2004)
		Leaves	GC–MS	Bajpai and Kang (2011b)
349	Pyridine	Leaves	GC–MS	Bajpai et al. (2009)
		Leaves	GC–MS	Bajpai and Kang (2011b)
350	Pyrrolidine	Leaves	GC–MS	Bajpai et al. (2009)
		Leaves	GC–MS	Bajpai and Kang (2011b)
	*Sterols*			
351	β-Sitosterol	Leaves	MS, IR	Beckmann and Schuhle (1968)
		Twigs	LST, MP, IR	Hayashi et al. (1969)
		Leaves	MP, UV, MS, NMR	Duan et al. (2009)
		Branches and stems	N/A	Zeng et al. (2013)
352	Campesterol	Twigs	LST, MP, IR	Hayashi et al. (1969)
353	Stigmasterol	Twigs	LST, MP, IR	Hayashi et al. (1969)
	*Steroids*			
354	Campest-4-en-3-one	Twigs	IR, UV, NMR, MP	Hayashi et al. (1969)
355	Stigmast-4-en-3-one	Twigs	IR, UV, NMR, MP	Hayashi et al. (1969)
356	Stigmast-4-22-dien-3-one	Twigs	IR, UV, NMR, MP	Hayashi et al. (1969)
357	5α-Stigmastan-3,6-dione	Twigs	IR, ORD,MS, NMR	Hayashi et al. (1969)
	*Sugars*			
358	Fructose	Leaves	PC	Kariyone et al. (1958)
		Leaves	PC	Hida et al. (1962)
359	Galactose	Leaves	PC	Kariyone et al. (1958)
360	Glucose	Leaves	PC	Kariyone et al. (1958)
		Leaves	PC	Hida et al. (1962)
361	Sucrose (Saccharose)	Leaves	PC	Kariyone et al. (1958)
		Leaves	PC	Hida et al. (1962)
362	α-*D*-Fructofuranoside	Branches and stems	N/A	Zeng et al. (2013)

*EA* elemental analysis, *FID* flame ionization detection, *GC* gas chromatography, *GLC* gas–liquid chromatography, *GC–MS* gas chromatography mass spectrometry, *IR* infrared spectroscopy, *LST* Liebermann and Salkowski color test, *MS* mass spectrometry, *MP* melting point, *NMR* nuclear magnetic resonance, *OR* optical rotation, *PC* paper chromatography, *PPC* paper partition chromatography, *S* standard [comparison of unknown with standard compound (s)], *TLC* thin layer chromatography, *UV* ultra violet to visible spectroscopy, *N/A* not available

^a,b,c,d^Compound names labelled with the same letter may refer to the same compound

The longevity of *M. glyptostroboides* may make this species a molecular window into the ancient world. Technological improvements allowing for characterization of modified and original natural products from fossil material, have consequently lead to characterization of such compounds from fossil leaves from *M. glyptostroboides*. These compounds which are included in the current review (Table 2) (Zhao et al. 2007) include two natural products reported both from fresh leaves and fossil leaves (Table 1 and 2) (Fujita 1990; Zhao et al. 2007). In line with this, correlations to recent identifications of natural products and modified derivatives thereof from well preserved fossil *M. glyptostroboides* originating from the Miocene era are discussed.

**Table 2:** Compounds identified from fossil leaves from *Metasequoia glyptostroboides* Hu et Cheng

No.	Substances alphabetically according to group	Part of the tree	Method of identification	References
	*Alcohols*			
1	2,3-Dimethyl-3-buten-2-ol	Fossil leaves	GC–MS	Zhao et al. (2007)
2	2-Methyl-Cyclopentanol	Fossil leaves	GC–MS	Zhao et al. (2007)
3	2-Hexanol	Fossil leaves	GC–MS	Zhao et al. (2007)
4	2-Heptanol	Fossil leaves	GC–MS	Zhao et al. (2007)
5	2-Hexyl-1-decanol	Fossil leaves	GC–MS	Zhao et al. (2007)
6	(*E*)-2-undecen-1-ol	Fossil leaves	GC–MS	Zhao et al. (2007)
7	2-methyl-3-(1-metylethenyl)-cyclohexanol	Fossil leaves	GC–MS	Zhao et al. (2007)
	*Aldehydes*			
8	2-(*E*)-hexenal	Fossil leaves	GC–MS	Zhao et al. (2007)
9	Decanal	Fossil leaves	GC–MS	Zhao et al. (2007)
	*Alkanes*			
10	Pentadecane	Fossil leaves	GC–MS	Zhao et al. (2007)
11	Hexadecane	Fossil leaves	GC–MS	Zhao et al. (2007)
12	2,6,10,14-tetramethyl-hexadecane	Fossil leaves	GC–MS	Zhao et al. (2007)
13	Heptadecane	Fossil leaves	GC–MS	Zhao et al. (2007)
14	Octadecane	Fossil leaves	GC–MS	Zhao et al. (2007)
15	Nonadecane	Fossil leaves	GC–MS	Zhao et al. (2007)
16	Eicosane (Icosane)	Fossil leaves	GC–MS	Zhao et al. (2007)
17	Heneicosane	Fossil leaves	GC–MS	Zhao et al. (2007)
18	Docosane	Fossil leaves	GC–MS	Zhao et al. (2007)
19	Tricosane	Fossil leaves	GC–MS	Zhao et al. (2007)
20	Tetracosane	Fresh leaves	GC–MS	Fujita (1990)
		Fossil leaves	GC–MS	Zhao et al. (2007)
21	Pentacosane	Fresh leaves	GC–MS	Fujita (1990)
		Fossil leaves	GC–MS	Zhao et al. (2007)
22	1,2-Dimethylcyclopentane	Fossil leaves	GC–MS	Zhao et al. (2007)
	*Esters*			
23	Dibutyl phthalate^a^	Fossil leaves	GC–MS	Zhao et al. (2007)
24	Diisobutyl phthalate^a^	Fossil leaves	GC–MS	Zhao et al. (2007)
25	Bis (2-ethylhexyl) phtalate^a^	Fossil leaves	GC–MS	Zhao et al. (2007)
	*Furans*			
26	Dibenzofuran	Fossil leaves	GC–MS	Zhao et al. (2007)
	*Ketones*			
27	1-(methylphenyl)-ethanone	Fossil leaves	GC–MS	Zhao et al. (2007)
28	3-(*E*)-Penten-2-one	Fossil leaves	GC–MS	Zhao et al. (2007)
29	4-Hydroxy-4-Methyl-2-pentanone	Fossil leaves	GC–MS	Zhao et al. (2007)
30	1-(-Naphthalenyl)-ethanone	Fossil leaves	GC–MS	Zhao et al. (2007)
31	1,7,7-trimethyl-bicyclo2.2.1hetpan-2-one	Fossil leaves	GC–MS	Zhao et al. (2007)
32	6,8-Dioxabicyclo [3.2.1] octane	Fossil leaves	GC–MS	Zhao et al. (2007)
33	Benzophenone	Fossil leaves	GC–MS	Zhao et al. (2007)
34	Tetrahydro-3,6-dimethyl-2H-pyran-2-one	Fossil leaves	GC–MS	Zhao et al. (2007)
	*Fatty acids and their derivatives*			
35	Dodecanoic acid, methyl ester	Fossil leaves	GC–MS	Zhao et al. (2007)
36	Formic acid octyl ester	Fossil leaves	GC–MS	Zhao et al. (2007)
37	Hexadecanoic acid methyl ester	Fossil leaves	GC–MS	Zhao et al. (2007)
38	Octadecanoic acid methyl ester	Fossil leaves	GC–MS	Zhao et al. (2007)
39	Tetradecanoic acid methyl ester	Fossil leaves	GC–MS	Zhao et al. (2007)
	Aromatic hydrocarbons			
40	Anthracene	Fossil leaves	GC–MS	(Zhao et al. 2007)
41	Naphthalene	Fossil leaves	GC–MS	Zhao et al. (2007)
42	1-Methyl-naphthalene	Fossil leaves	GC–MS	Zhao et al. (2007)
43	2-Methyl-naphthalene	Fossil leaves	GC–MS	Zhao et al. (2007)
44	Retene	Fossil leaves	GC–MS	Zhao et al. (2007)
	*Isocyanate*			
45	Isocyanato-cyclohexane	Fossil leaves	GC–MS	Zhao et al. (2007)
	*Terpenoids*			
	I. Monoterpenoids			
46	*L*-(−)-menthol	Fossil leaves	GC–MS	Zhao et al. (2007)
	II. Diterpenoids and their derivatives			
47	2,6,10-Trimethyl-hexadecane	Fossil leaves	GC–MS	Zhao et al. (2007)
	III. Triterpenoids			
48	Squalene	Fossil leaves	GC–MS	Zhao et al. (2007)
	*N-heterocycles*			
49	2,3-Dimethyl-*N*-phenylpyrrolidine	Fossil leaves	GC–MS	Zhao et al. (2007)
	*Sulphur-containing compounds*			
50	4-Hydroxybenzenesulfonic acid	Fossil leaves	GC–MS	Zhao et al. (2007)
51	1,2-Benzisothiazole	Fossil leaves	GC–MS	Zhao et al. (2007)

*GC–MS* gas chromatography mass spectrometry

^a^These compounds are known plasticizers and could as such be artefacts

## Brief History

*Metasequoia* was first described as a new extinct genus in 1941 by the Japanese paleobotanist Shigero Miki (1901–1974) (Miki 1941). He based his work on field samples of fossil remains from Japan, which he identified himself. Based on these observations Miki described two new species that were different from Sequoia, but with some common features, and renamed two published species previously ascribed to *Sequioa*. The first of these species was *Sequoia disticha Heer*, which was described in 1876 by Oswald Heer (1809–1883), a Swiss pioneer in paleobotany, based on field samples of fossil remains collected by a Swedish expedition to Svalbard in 1872–1873 (Heer and Nordenskiöld 1876). Five decades later the second species, Sequoia japonica Endô, was described in 1936 by the Japanese paleontologist Seidô Endô based on field samples from Korea and Japan (Endô 1936). The name of the new genus means “resemble a *Sequoia*”, and acknowledges the fact that the two genera *Sequoia* and *Metasequoia* resemble each other.

During the early 1940′s a series of events in southeast China led to the sensational discovery of a living species of *Metasequoia*. At the centre of the events is a large deciduous tree, in the small village of Moudao in western Szechuan (Sichuan), locally known as “shui-sha” or water fir in English (Hsueh 1985; Hu 1948a). The story of the collection of specimens and identification of the tree covers seven years from 1941 to 1948, and a complete summary of events and the people involved is beyond the scope of this article. An account of the discovery of *Metasequoia* was written by Hu in 1948 (Hu 1948a). The great interest and rapid accumulation of botanical knowledge necessitated a botanical review as early as 1952 (Florin 1952). Fifty years after the first description of the tree a special thematic issue of *Arnoldia* (Madsen 1998–1999) celebrated the event and a detailed review of the chronology of the history of *M. glyptostroboides* was written by Ma in 2003 (Ma 2003).

## Natural habitat and distribution of *M. glyptostroboides*

*M. glyptostroboides* is endemic to southeast China where the largest native population is found in the Shui-Hsa River valley, also called Xiaohe River Valley, in Zhonglu in Hubei Province (Wang et al. 2006). However, native trees have also been found in an estimated area of about 800–1000 km^2^ within eastern Chongqing municipality, western Hubei, and western Hunan Provinces (Bartholomew et al. 1983; Chu and Cooper 1950; Gressit 1953; Leng et al. 2007; Tang et al. 2011; Wang et al. 2006). In this region the tree occurs as a constituent of the Mixed Mesophytic Forest and grows at an altitude ranging from 800 to 1500 m. Because of this limited distribution, the declining number of individuals, the decreasing available habitat, together with low genetic diversity (Li et al. 2005), *M. glyptostroboides* is classified as endangered on The IUCN Red List of Threatened Species (Farjon 2013). The natural habitat of the tree is in the humid and warm lower mountain slopes with river and stream valleys. In the nearby city of Lichuan, 1083 m above sea level and approximately 60 km from Zhonglu, the monthly mean temperature varies from around 1.9 ^o^ C in January to 22.6 ^o^ C in August with an annual mean temperature of 12.7 ^o^ C. Rainfall is seasonal with a mean annual precipitation of 1319 mm, most of which (85 %) falls during the seven months from April to October (Tang et al. 2011). After discovering *M. glyptostroboides* as a living species, there was an intense effort to cultivate the tree throughout the world (Chu and Cooper 1950). The tree is highly adaptable and since 1948, *M. glyptostroboides* has been successfully grown in nearly 50 countries in Asia, Africa, Europe and America (Ma 2007).

## Botanical description

**Fig. 1:**
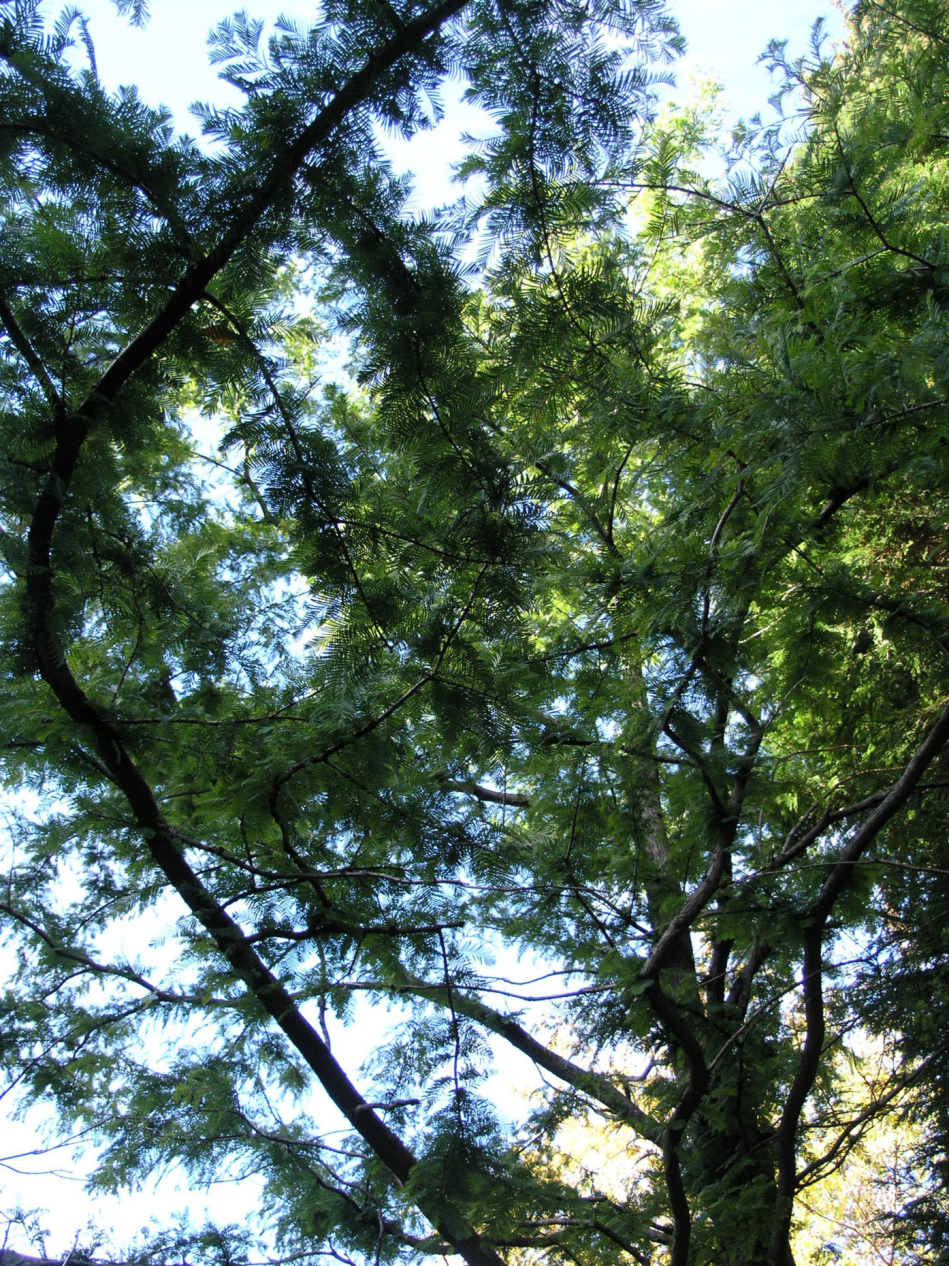
*Metasequoia glyptostroboides* grown in the Botanical Museum garden of University of Bergen, Bergen, Norway. Photo: Torgils Fossen

*M. glyptostroboides* is a large deciduous conifer that belongs to the family Cupressaceae (Fig. 1) and is the only living species in the genus. It is a fast growing tree that can reach a height of 45 m and 2.2 m in diameter (Ma 2007). *M. glyptostroboides* has a pyramidal shape when young, but can develop a more rounded shape with age. The bark is reddish brown in the early stage, and becomes darker and more greyish over time, with vertical furrows and armpits under the branches. The branchlets are up to about 7.5 cm long and usually arranged distichously with up to 50–60 leaves. The bright green opposite linear leaves provide foliage of feathery texture in mid-spring. During autumn the colour changes to orange, yellow and red-brown before the foliage falls off in wintertime. *M. glyptostroboides* is monoecious, with both male (pollen) and female cones growing on different branches of the same tree. The trees can in general produce cones when they are 9–15 m high, while pollen cones are produced when the tree attains a height of 18–27 m. Pollen cones are pendulous (5–6 mm long), and are produced mid-June, pollen forms in November, and is dispersed with wind in early spring, and is only produced in regions with relatively warm climates. The cones are globose to ovoid (1.5–2.5 cm long) with 16–28 scales in opposite pairs in four rows. The cone is produced early in July, but fertilization occurs in June the following year. The seeds mature 4–5 months after fertilisation (Li 1998/1999).

## Natural products from *Metasequoia glyptostroboides*

**Fig. 2:**
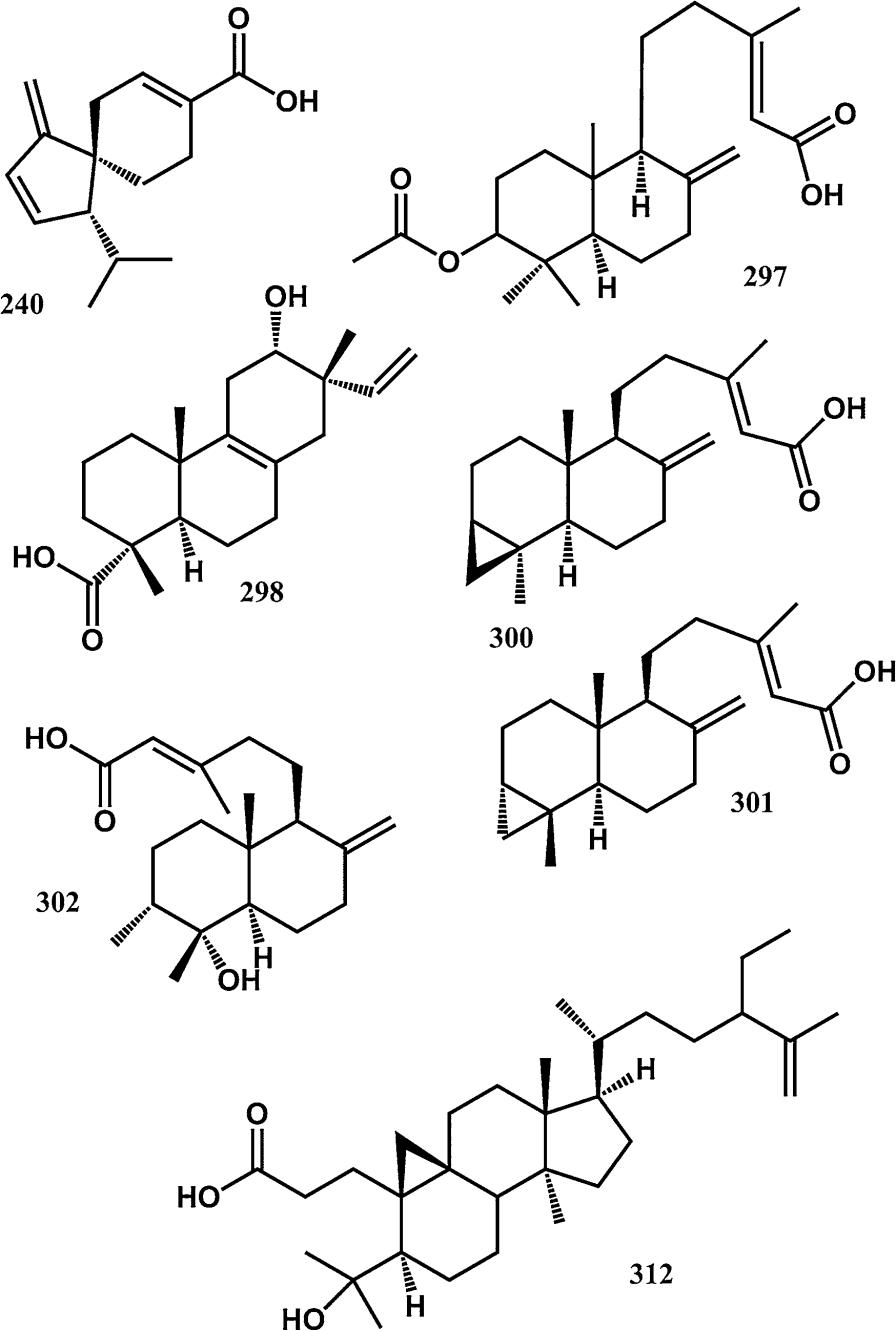
Novel natural products isolated from *Metasequoia glyptostroboides*. I. Terpenoids

**Fig. 3:**
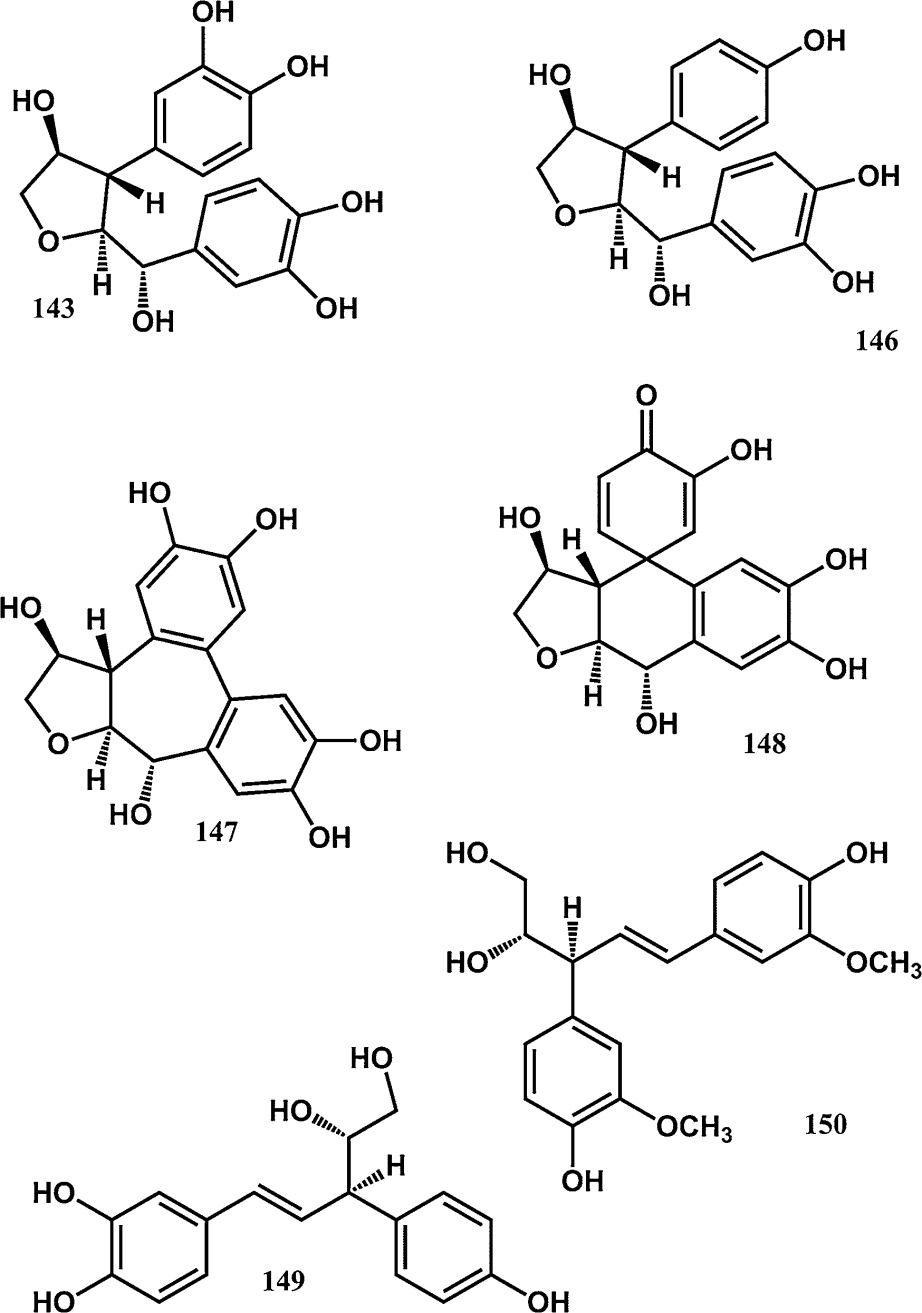
Novel natural products isolated from *Metasequoia glyptostroboides*. II. Norlignans

**Fig. 4:**
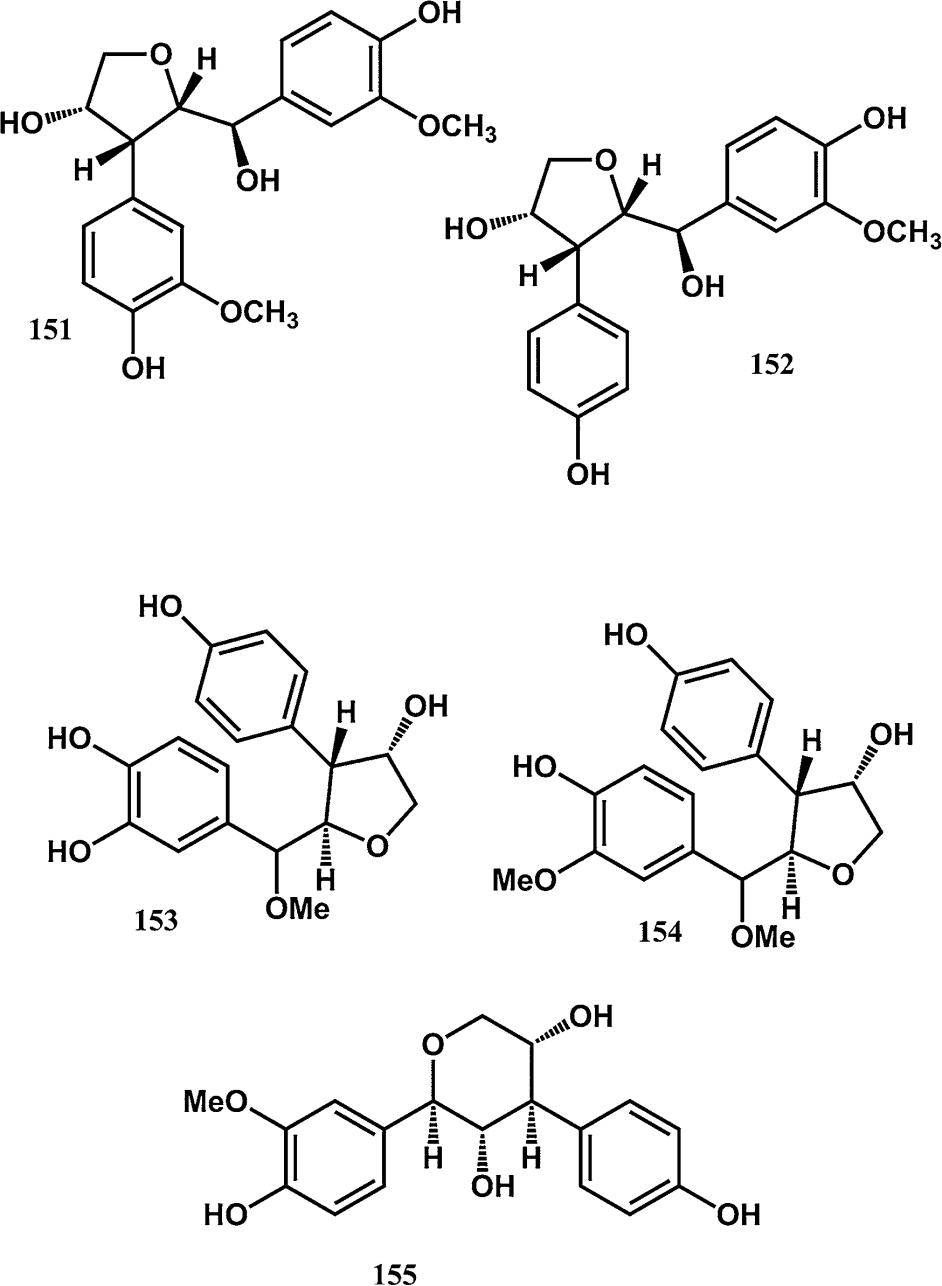
Novel natural products isolated from *Metasequoia glyptostroboides*. III. Norlignans (continued)

**Fig. 5:**
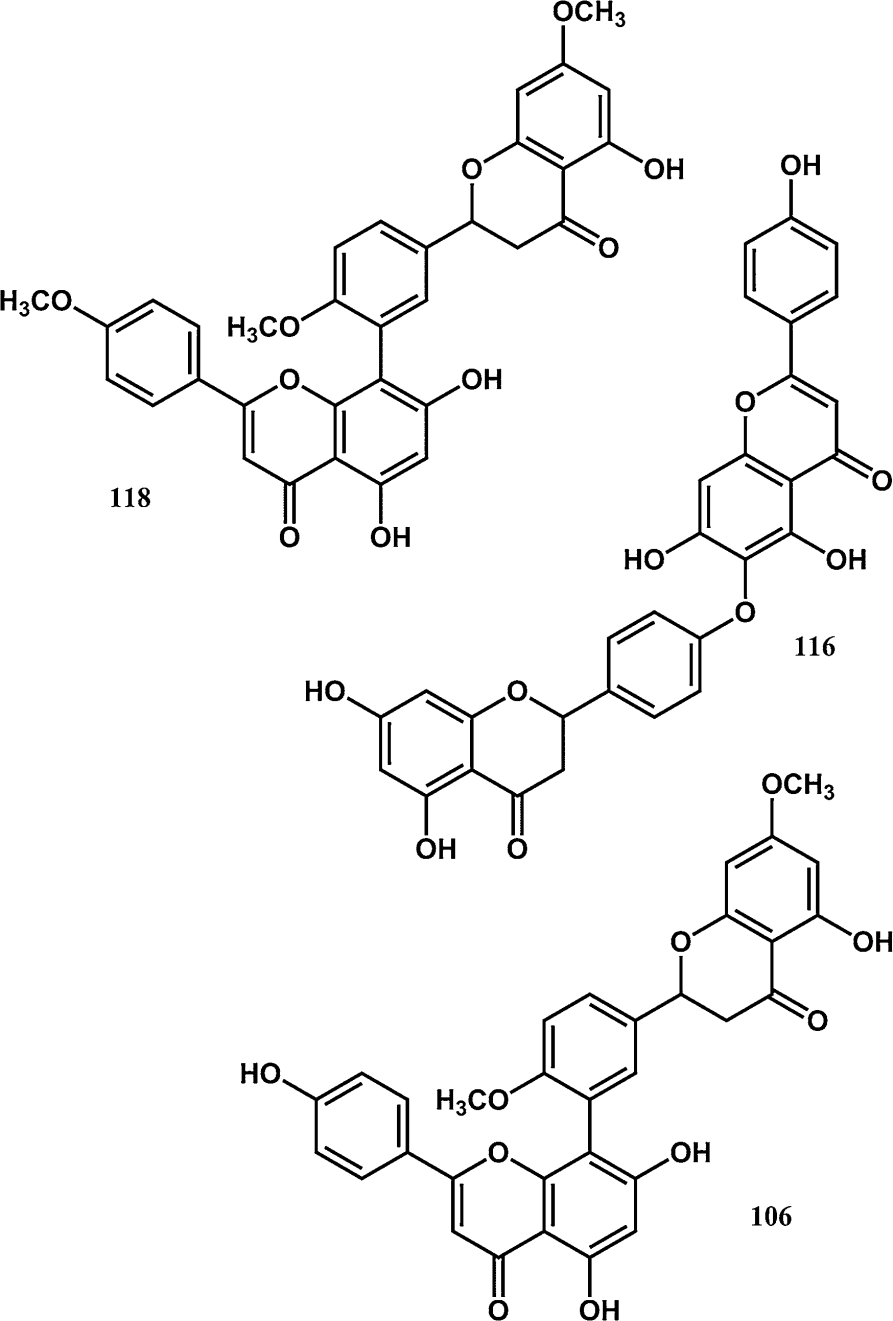
Novel biflavonoids characterized from *Metasequoia glyptostroboides*

**Fig. 6:**
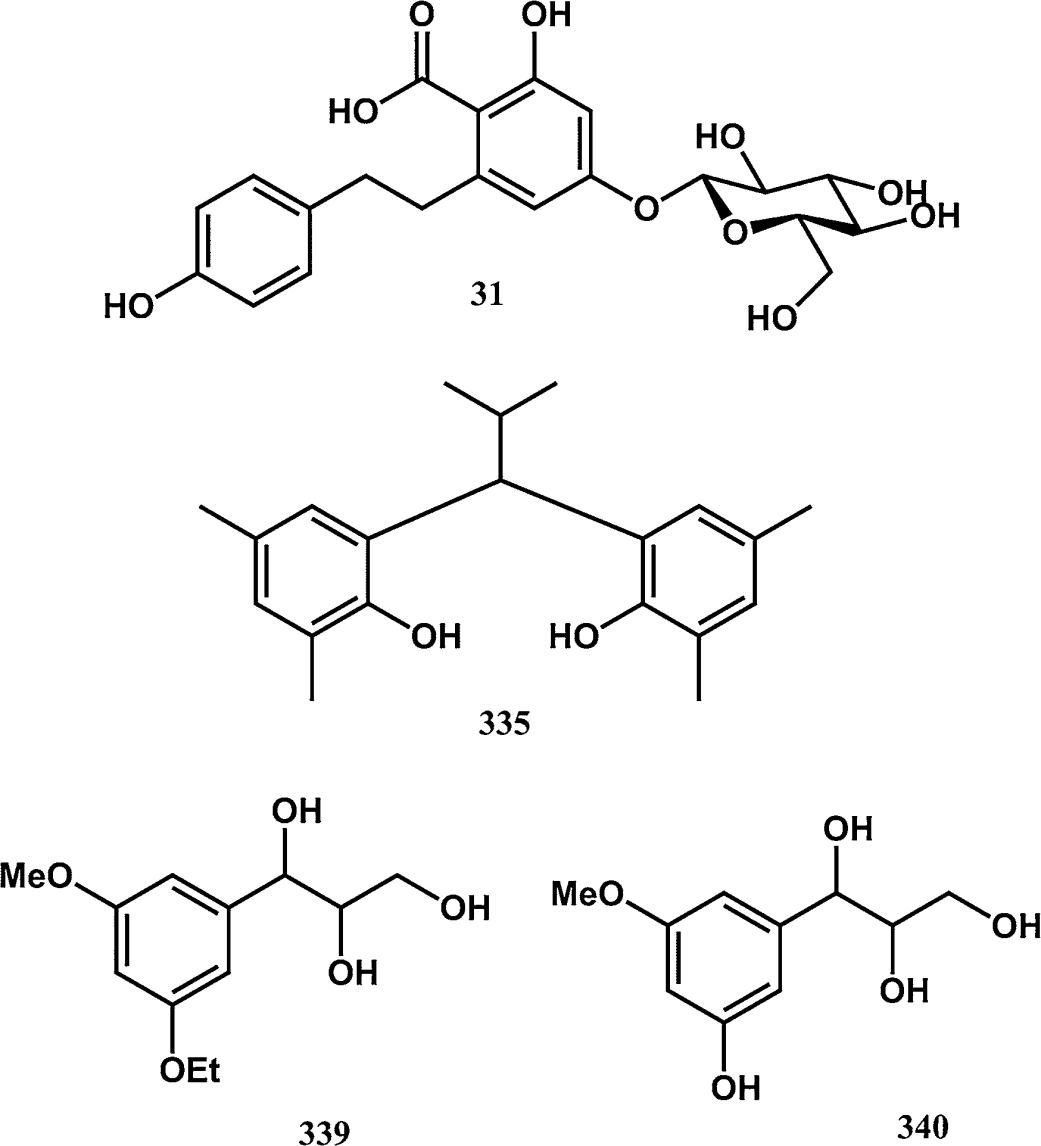
Novel natural products isolated from *Metasequoia glyptostroboides*. V. Other phenolic compounds

**Fig. 7:**
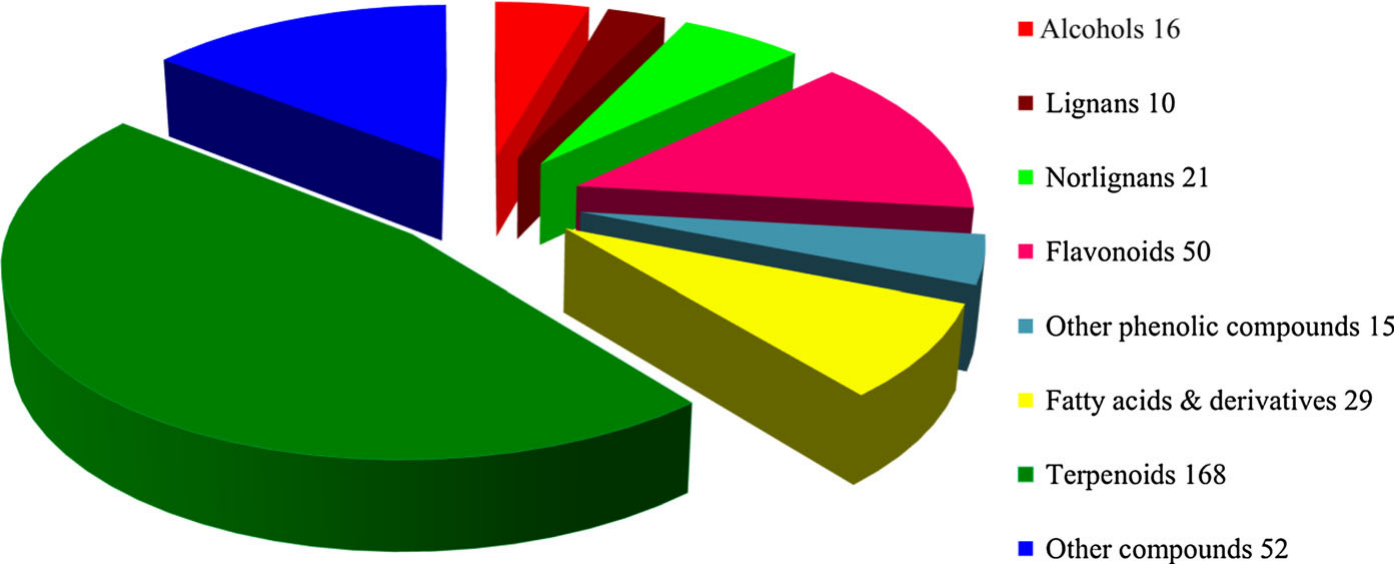
Classes of natural product characterized from *Metasequoia glyptostroboides*

**Fig. 8:**
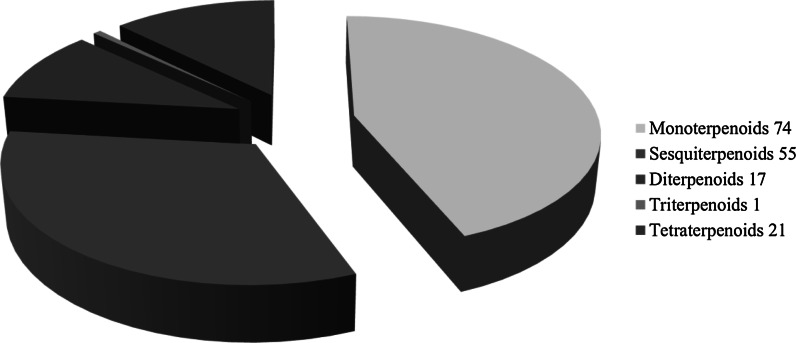
Overview of groups of terpenoids characterized from *Metasequoia glyptostroboides*

**Fig. 9:**
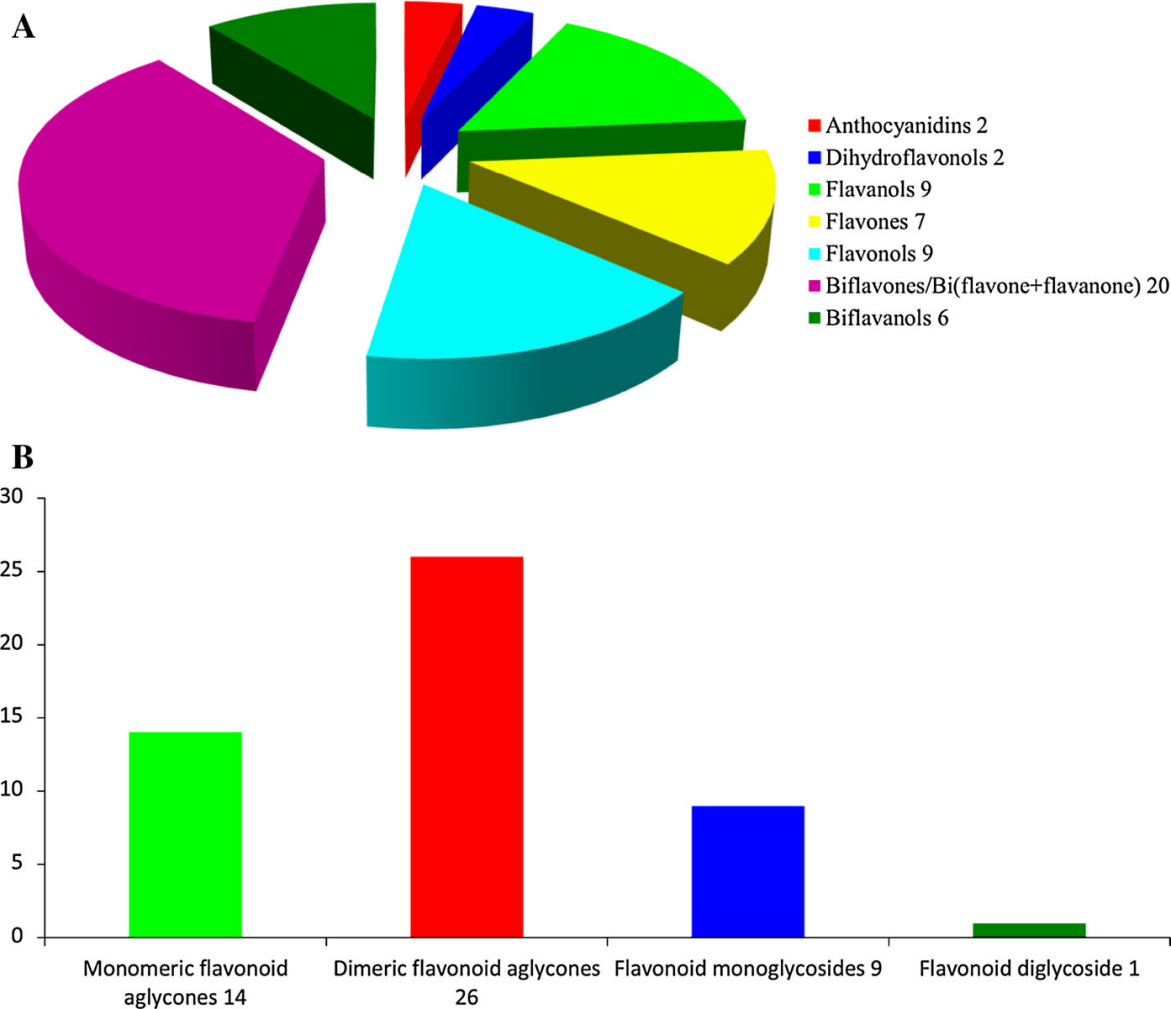
Overview of flavonoid classes characterized from *Metasequoia glyptostroboides* based on type of aglycone (**a**) and extent of glycosylation (**b**)

To assist current and future researchers with interests in the vast number of natural products from *M. glyptostroboides*, all compounds hitherto reported from this species are systematized for the first time in Table 1, according to compound class. The information provided also includes from which part of the tree the compounds have been detected, as well as the methods used for identifications in each instance where such information is available. Approximately 362 natural products have been characterized from *M. glyptostroboides* (Table 1). The majority of these compounds have been characterized from the leaves, although seeds, branches, heartwood and bark have also been analyzed (Table 1). Twenty-six natural products were unique to *M. glyptostroboides* at the time they were characterized (Figs. 2, 3, 4, 5, 6). The structures of these novel compounds are shown in Figs. 2, 3, 4, 5, and 6. The compound classes, which include natural products specific to *M. glyptostroboides* are discussed in detail below. The various categories of natural products from this plant source are systematized in Figs. 7, 8, and 9.

## Characterization and structure elucidation

The majority of known compounds reported from *M. glyptostroboides* are relatively volatile, which may reflect the fact that the majority of samples from this plant source have been characterized by GC–MS. X-ray data have not been reported for any compound isolated from *M. glyptostroboides*. However, an increasing number of compounds have been characterized in detail at atomic resolution, mainly by using a combination of 2D NMR spectroscopy and MS (Table 1). Supporting structural information for a not insignificant minority of the characterized compounds has been achieved by using OR (for chiral compounds) and IR spectroscopy, as well as various forms of co-chromatography and MP determinations (Table 1).

## Terpenoids

Terpenoids comprise the largest group of natural products characterized from *M. glyptostroboides*. Until now, 168 different terpenoids have been reported from this plant source (Table 1 and Fig. 7). The majority of these compounds are monoterpenoids, of which 74 have been identified (Fig. 8). Conifers are known to be rich sources of monoterpenoids (Cvrkal and Janak 1959). All of these monoterpenoids are known from other plant sources, as is also the case for the 21 tetraterpenoids (carotenoids) and the single triterpenoid identified. Sesquiterpenoids comprise the second largest group of terpenoids identified from *M. glyptostroboides* counting 55 different structures (Fig. 8). One of these, namely (−)-acora-2,4(14),8-trien-15-oic acid (**240**) is specific to *M. glyptostroboides* (Fig. 2). Among the 17 diterpenoids reported, the six compounds 3β-acetoxy-8 (17),13 E-labdadien-15-oic acid (**297**), 12α-hydroxy-8,15-isopimaradien-18-oic acid (**298**), metasequoic acid A-C (**300–302**), and metaseglyptorin A (**312**) are specific to *M. glyptostroboides* (Fig. 2).

## Flavonoids

Flavonoids are the most important polyphenolic compounds synthesized by plants. According to Markham (1982) approximately 2 % of all carbon photosynthesized by higher plants are biosynthetically converted to flavonoids (Markham 1982). More than 10 000 different flavonoids have hitherto been reported (Tahara 2007). No less than 50 flavonoids have been reported from *M. glyptostroboides*, which means that they are one of the main groups of natural products characterized from this tree (Figs. 7, 9a). The majority of them are non-glycosylated monomeric (14) or dimeric (26) flavonoids (Fig. 9b). Nine flavonoid monoglycosides and one flavonoid diglycoside have been reported from *M. glyptostroboides*. The glycosylation positions in these compounds are restricted to the 3-, 7- and 3′-positions of the aglycones (Table 1). Glucose and rhamnose are the only sugar units found in the flavonoid glycosides reported from *M. glyptostroboides*, where glucose is the predominant glycosyl unit (Table 1). Acylated flavonoids have hitherto not been identified from this species. The flavonoids most characteristic for *M. glyptostroboides* are dimers of either two flavone units or a flavone and a flavanone unit (Table 1 and Fig. 5). Three such compounds, namely 2, 3-dihydroamentoflavone-7″,4′″-dimethylether (**106**), 2,3-dihydrohinokiflavone (**116**) and 2,3-dihydrosciadopitysin (**118**) were discovered in nature for the first time from this species (Fig. 5). Moreover, an anticancer drug based on one of these compounds (dihydrohinokiflavone) isolated from *M. glyptostroboides* has been patented (Jung et al. 2004).

## Lignans and norlignans

The largest population of compounds specific to *M. glyptostroboides* belongs to the norlignans. Lignans and norlignans comprise classes of phenylpropanoid-derived natural products with abundant occurrence in nature (Suzuki and Umezawa 2007). Lignans are dimeric phenylpropanoids where the monomers are linked at the central carbon (C8) (Suzuki and Umezawa 2007). Norlignans are naturally occurring phenolic compounds based on a diphenylpentane carbon skeleton consisting of a phenyl–ethyl unit linked to a phenyl-propyl unit. Lignans are widely distributed within the plant kingdom (Suzuki and Umezawa 2007), while norlignans, on the other hand, are mainly found in conifers and monocotyledons (Suzuki and Umezawa 2007).

While some lignans are already established as active principles of anticancer drugs such as podophyllotoxines (Stahelin and von Wartburg 1991), there is also an increased recent interest in research on norlignans with significant anticancer activity such as agatharesinol acetonide isolated from *Sequoia* (Zhang et al. 2005). Altogether 10 lignans have been reported from *M. glyptostroboides* (Table 1). All of these compounds are known from other plant sources. The biosynthetic pathways of the norlignans of *M. glyptostroboides* appear, however, to be more unique to this species. Among the 21 norlignans characterized from this plant source (Table 1), the majority of the compounds, namely hydroxyathrotaxin (**143**), hydroxymetasequirin A (**147**), and metasequirin A-I (**146, 148–155**) are unique to *M. glyptostroboides* (Figs. 3, 4).

## Other aromatic compounds specific to *M. glyptostroboides*

Four further aromatic natural products unique to *M. glyptostroboides* deserve particular attention. The symmetric natural product metaseol (**335**), isolated from the root bark, belongs to the diphenylmethanes, a relatively rare class of natural product (Nakatani et al. 1991). Metaseol has only been detected in *M. glyptostroboides* and is the first and only symmetric diphenylmethane ever isolated from any natural source. The two new phenylpropanoids 7-(3-ethoxy-5-methoxyphenyl)propane-7,8,9-triol (**339**) and 7-(3-hydroxy-5-methoxyphenyl) propane-7,8,9-triol (**340**) (Fig. 6) isolated from branches and stems of *M. glyptostroboides* exhibited mild cytotoxic activity against A549 and Colo 205 cell lines (Zeng et al. 2012). 6-Carboxydihydroresveratrol-3-glucoside (**31**) is the only stilbenoid (bibenzyl) derivative hitherto reported from *M. glyptostroboides* (Nguyen et al. 2014). Bibenzyl aglycones with carboxylic substituents have a restricted occurrence in nature. The fact that these compounds have mainly been found in species belonging to the oldest lineages of plant families like the fern *Hicriopteris glauca* (Fang et al. 2012), Liverworts (Pryce 1971; Pryce 1972; Valio et al. 1969) and algae (Huneck and Pryce 1971) indicate that these compounds may be biogenetic precursors of modern plant stilbenoids, with the COOH group being a biogenetic archaicism (Nguyen et al. 2014).

## Temperature and sunlight conditions –potential influential factors on secondary metabolite synthesis

Reports on natural products from *M. glyptostroboides* available in current literature have been conducted on trees growing at quite a few rather different localities, including several European and Asian countries, including China (Dong et al. 2011), South Korea (Bajpai and Kang 2010a, 2011a, b; Bajpai et al. 2007a, b, 2009, 2010, 2014a; Duan et al. 2009), Japan (Fujita 1990; Hayashi et al. 1969; Ida 1981a, b; Nakatani et al. 1991), Poland (Czeczuga 1987; Krauze-Baranowska 2004), Germany (Beckmann and Geiger 1968; Beckmann et al. 1971; Beckmann and Schuhle 1968; Braun and Breitenbach 1977), France (Mongrand et al. 2001) and Norway (Nguyen et al. 2014). The natural products isolated from *M. glyptostroboides* grown in Norway were mainly different from those reported from the tree grown at other localities (Nguyen et al. 2014). This may be accounted for by the fact that the sunlight conditions (particularly the day length) in the growth season in Norway are quite different from that of other locations from which plant material has been analyzed. Temperature and sunlight conditions are known to be important parameters for the secondary metabolism of plants (Radušienė et al. 2012). However, it should be mentioned that Northern Europe, as far north as Svalbard, was part of the natural habitat of *M. glyptostroboides* until the Miocene era (23–5 million years ago) from which fossils of this species were discovered for the first time in 1876 (but originally incompletely identified) (Heer and Nordenskiöld 1876). To date, however, no comprehensive systematic study has been performed to investigate the influences of any growth conditions or locality on secondary metabolite production of *M. glyptostroboides*.

## Natural products of *Metasequoia glyptostroboides* –a molecular window into the Cretaceous era

*Metasequoia* is presumed to have evolved in eastern Russia during the early Late Cretaceous period, around 100 million years ago as the earliest dawn redwood fossils were reported from this region (Yang 1998/1999). The unique potential of the tree as a source of bioactive constituents is founded on the fact that it seems to have survived unchanged since the Cretaceous era. Since then, its unusually successful molecular defense system has resisted the attacks of millions of generations of pathogens. Unlike fossils, where the original molecules may be fragmentary at best, if present at all (Schweitzer et al. 2009), living fossils like *M. glyptostroboides* may provide a detailed, intact, high-resolution system from which ancient natural products can be uncovered and characterized. However, already at a very early stage after its discovery, doubt was cast about whether or not *M. glyptostroboides* had remained unchanged at the molecular level –or even if the present species could reasonably be named identically to a species existing in the Cretaceous era (Schopf 1948). The predominant view was that in fossils of plants, only the morphology was preserved, whereas the original molecules were lost (Calvin 1969). Until recently, detection or any identification of the original natural products of fossil material of any species appeared to be unlikely. However, recent development in analytical technology has made it possible to identify at least fragments of the original molecules, modified natural products or even unmodified natural products from well preserved fossils dating back as far as to the Cretaceous era (Bern et al. 2009; Schweitzer 2004; Schweitzer et al. 1997). As a consequence, Zhao et al. (2007) succeeded in identifying 51 different compounds from fossil *M. glyptostroboides* excavated at Svalbard, Norway, dating from the Miocene era (23–5 million years ago) (Table 2) (Zhao et al. 2007). Although the majority of these compounds were considered to be modified natural products, which may, however, in some instances had kept their original core structures (such as squalene and retene), two of these compounds, namely the hydrocarbons tetracosane and pentacosane, have also been identified from fresh plant material (Tables 1 and 2) (Fujita 1990; Zhao et al. 2007). Hydrocarbons are among the few natural products with sufficient expectable life time to be discovered intact in fossilized material which has been preserved over a time scale of millions of years (Calvin 1969). When keeping in mind that the growth conditions may influence the biosynthesis of natural products of *M. glyptostroboides* as indicated by Nguyen et al. (2014) (Nguyen et al. 2014), further compounds reported by Zhao may be either compounds with intact core structure or surviving original natural products from the relatively warm Miocene era, when the natural habitat of the tree included Svalbard in the far north. Very recently, an intact and significantly older piece of *Metasequoia* wood buried deeply in a kimberlite pipe that intruded northwestern Canada’s Slave Province 53.3 ± 0.6 million years ago was discovered (Wolfe et al. 2012). Initial comparative IR spectral analysis of this intact 53 million year old wood and amber of *Metasequoia* with fresh wood from present *M.* *glyptostroboides* gave similar results, strongly indicating that the tree has remained unchanged for millions of years at the molecular level (Wolfe et al. 2012). Attempts to recover DNA from well preserved fossilized *Metasequoia* needles encapsulated in amber have hitherto been unsuccessful (Yang 1998/1999). However, it may be possible that the 53 million year old intact *Metasequoia* wood recently discovered could contain intact DNA or sufficiently large fragments thereof required for a direct comparison with DNA of the present *M. glyptostroboides*.

## Biological and pharmacological effects of substances and extracts of *M. glyptostroboides*

An increasing number of studies of various biological activities and medicinal applications of the title plant have been reported in current literature. These include studies performed on pure compounds, as well as extracts, and applications as plant medicines. Several recent patents exploiting substances or extracts of *M. glyptostroboides* visualize the increased commercial potential of medicinal applications based on the bioactive constituents from this species. (Ding 2003; Jung et al. 2004; Lee et al. 2009; Wu 2009). The different types of biological activities reported in current *Metasequoia* literature are treated in separate paragraphs below.

## Antioxidant activity

Antioxidant activity, as well as radical scavenging activity has been determined for both extracts and pure compounds from *M. glyptostroboides*. Bajpai et al. (2009) tested the antioxidant activity of the essential oil and various organic extracts (n-hexane, chloroform, ethyl acetate and methanol) of *M. glyptostroboides*. DPPH was used to identify antioxidant activity. The study revealed that essential oil and ethyl acetate extracts showed higher or similar antioxidant activity compared to the standards, butylated hydroxyanisole and ascorbic acid. This might be accounted for by the high total phenolic content in the ethyl acetate extracts (Bajpai et al. 2009). Chen et al. (2014) reported significant DPPH radical, superoxide anion radical, and hydroxyl radical scavenging capacity, total antioxidative capacity, lipid peroxidation inhibitory activity, and metal ions chelating capacity of chromatographic fractions derived from bark extracts of *M. glyptostroboides*. The observed activities were correlated with the proanthocyanidin content of the active fractions isolated (Chen et al. 2014).

The DPPH scavenging activity of the pure compound 6-carboxydihydroresveratrol-3-*O*-β-glucopyranoside isolated from *M. glyptostroboides* was significant, though the IC_50_ value was approximately 11-fold higher than the reference compound gallic acid (Nguyen et al. 2014). Hinokiflavone, a biflavone which occurs in leaves of *M. glyptostroboides*, has been identified as a potent antioxidant using hyphenated HPLC-DPPH (Zhang et al. 2011). The compound used for these studies was, however, not isolated from *M. glyptostroboides*.

## Arachidonic acid metabolism inhibition

Arachidonic acid metabolites play important roles in disease conditions such as inflammation and development of cancer (Hyde and Missailidis 2009). Therefore, there is an increasing interest in discovering inhibitors of key enzymes of the arachidonic acid cascade reaction, such as 15-lipoxygenase (Gillmor et al. 1997; Samuelsson et al. 1987). The dihydrostilbenoid glucoside 6-carboxydihydroresveratrol-3-*O*-β-glucopyranoside, a compound specific to *M. glyptostroboides*, proved to be a significant inhibitor of 15-lipoxygenase with IC_50_ at a comparable level to the standard inhibitor quercetin (Nguyen et al. 2014).

## Antibacterial effect

There is a continuous need for the discovery of novel antibiotics, due to the observed development of bacterial resistance to the antibiotics presently known. Because *M. glyptostroboides* has resisted the attack of millions of generations of pathogens, apparently without changing, the tree may be a promising source of natural products with antibiotic activity. Indeed, significant antibiotic activity towards several types of bacteria has been reported for extracts, as well as for pure compounds derived from this species.

Bajpai et al. (2007a) identified 59 compounds from the floral cone of *M. glyptostroboides*, which mainly contained oxygentated mono- and sesquiterpenes and the corresponding hydrocarbons. These compounds together with the complete methanol extract and methanol derived sub fractions were tested for antimicrobial effect against eleven different food spoilage and foodborne bacterial strains, four gram-positive bacteria and seven gram-negative bacteria. The essential oil, methanol extracts and various organic sub-fractions exhibited significant potential for antibacterial activity. The study indicated that mediated essential oils and extracts from *M. glyptostroboides* can be applied as natural preservatives or flavouring additives in the food industry to control spoilage and foodborne pathogenic bacteria which cause severe destruction of food (Bajpai et al. 2007a). Very recently, Bajpai et al. (2014a, b) reported anti-listeria activity of essential oils of *M. glyptostroboides*. The anti-listerial activity of essential oils of *M. glyptostroboides* acted synergistically with the peptide antibiotic nisin (Bajpai et al. 2014b).

The observed antibacterial activity of extracts derived from *M. glyptostroboides* may be rationalized by the fact that several pure compounds with significant antibacterial activity have been isolated from this plant source. Metaseol, a compound specific to *M. glyptostroboides*, exhibited potent antibacterial activity against *Bacillus subtilis* and *Escherichia coli* (Nakatani et al. 1991). Two abietane type diterpenoids, sugiol and taxodone, isolated from the ethyl acetate cone extract from *M. glyptostroboides*, proved to have antibacterial effect against several foodborne pathogenic bacteria, which may cause destruction and reduce the quality of food. Both studies showed that gram-positive bacteria were more sensitive to sugiol and taxodone than gram-negative bacteria. Sugiol exhibited higher antibacterial activity compared to the standard streptomycin in regard to gram-positive bacteria. Taxodone, on the other hand, exhibited lower antibacterial activity than the standard streptomycin. However both compounds inhibited gram-positive bacteria to some extent. The minimum inhibitory concentration (MIC) and minimum bactericidal concentration (MBC) for sugiol against foodborne pathogens were lower than for taxodone. The MIC is determined by the lowest concentration of the compound that does not show any growth of the test organism. MBC is defined as the complete absence of growth of bacterial colonies on the agar surface in the lowest concentration of sample. MIC for sugiol and taxodone varied from 62.5 to 250 μg/ml and 250–1000 μg/ml against different foodborne pathogens while MBC varied from 125 to 250 μg/ml and 250–2000 μg/ml, respectively. Similar antibacterial effects have also been detected for taxoquinone (Bajpai et al. 2010). The findings indicate that sugiol, taxodone and taxoquinone could be possible candidates for application in the food industry for the control of foodborne pathogens. Such potential applications would, however, require further studies on the safety and toxicity of these compounds (Bajpai and Kang 2010a, 2011a; Bajpai et al. 2010).

## Antifungal and antidermatophytic effects

The essential oil and various organic extracts (hexane, chloroform, ethyl acetate and methanol) of *M. glyptostroboides* have shown potential antidermatophytic effect against infectious fungal pathogens of the skin. They also inhibit some fungal spore germination at certain concentrations. Essential oils and extracts could therefore be used as a source of new antidermatophytic agents to control superficial human fungal infection (Bajpai et al. 2009). Bajpai and Kang have reported that the essential oil of *M. glyptostroboides* leaf has a moderate to high antifungal activity against seven different plant pathogenic fungal species namely *Botrytis cinerea* KACC 40573, *Rhizoctonia solani* KACC 4011, *Fusarium oxysporum* KACC 41083, *Sclerotinia sclerotiorum* KACC 41065, *Colletotrichum capsici* KACC 40978, *Fusarium solani* KACC 41092 and *Phytophthora capsici* KACC 40157. The results from the study also show that methanol, ethyl acetate and chloroform leaf extracts have strong antifungal activity against the tested plant pathogens. These findings indicate that the extracts and oil of *M. glyptostroboides* could be considered as potential antifungal agents to control several plant pathogenic fungi causing severe diseases in food, crops and vegetables (Bajpai and Kang 2010b).

Studies on antifungal activity of pure compounds isolated from *M. glyptostroboides* are hitherto limited to a few studies on diterpenoids. These include three antifungal diterpenoids reported by Asahi and Sakan (1984) (Table 1, compounds 305–307) and the diterpenoid taxoquinone (Bajpai and Kang 2014). The latter compound exhibited significant antifungal activity against pathogenic isolates of several *Candida* species.

## Antiviral activity

In current literature, studies on antiviral activity of natural products isolated from *M. glyptostroboides* have hitherto only been performed on pure hinokiflavone. This dimeric flavonoid, isolated from *M. glyptostroboides*, exhibited antiviral activity against influenza viruses A and B (Miki et al. 2008). The mechanism at molecular level is based on the fact that hinokiflavone acts as an inhibitor of viral sialidase (also known as viral neuraminidase/exo-α-sialidase) (Miki et al. 2008), an enzyme which plays at least two important roles in the viral life cycle. These include the facilitation of virion progeny release and general mobility of the virus in the respiratory tract (von Itzstein 2007). The observed anti-influenza activity was amplified significantly when hinokiflavone was conjugated with sialic acid (Miki et al. 2008). Several identified antiviral natural products originate from the shikimic acid biosynthetic pathway (Andersen and Helland 1996; De Bruyne et al. 1999; Hayashi et al. 2003), which is also the case for hinokiflavone. The B-ring systems of this dimeric flavonoid, in addition to C-2, and C-2″ originate from this biosynthetic pathway. The observed antiviral activity of these compounds may be rationalized by the fact that the slightly modified shikimic acid derivative oseltamivir, which is the active constituent of the anti-influenza drug Tamiflu, possess its antiviral activity through inhibition of the influenza viral sialidase (von Itzstein 2007).

## Anticancer activity

Recently, analyses of anticancer activity of extracts and pure compounds derived from *M. glyptostroboides* have been published. Zeng et al. (2012) reported that five pure compounds specific to *M. glyptostroboides*, namely the norlignans metasequirin G-I (**153–155**; Fig. 4) and the phenylpropans 7-(3-ethoxy-5-methoxyphenyl)propane-7,8,9-triol (**339**) and 7-(3-hydroxy-5-methoxyphenyl) propane-7,8,9-triol (**340**) (Fig. 6), exhibited cytotoxic activity against A549 and Colo 205 cell lines with IC_50_ values within the range 50–100 µM (Zeng et al. 2012). The fact that an anticancer drug based on dihydrohinokiflavone isolated from *M. glyptostroboides* has been patented (Jung et al. 2004) should encourage exploitation of the anticancer potential of the multitude of structurally relatively similar biflavonoids identified in leaves of this species (Table 1).

## Protective effects on cerebral ischemia–reperfusion injury

Wang et al. (2004) reported that a mixture of flavonoids from *M. glyptostroboides* (referred to as total flavonoids) exhibited protective effects on cerebral ischemia–reperfusion injury in rats (Wang et al. 2004). This is in agreement with the previous findings that intake of flavonoid-rich food has been reported to significantly improve coronary circulation in healthy human adults (Shiina et al. 2009).

## Other medicinal applications

As a medicinal plant *M. glyptostroboides* is a constituent of a plant medicine used for treatment of diabetes (Ding 2003) and has also applications in traditional Chinese medicine (TCM) (Wu 2009). Medicinal compositions for skin care have been prepared from *M. glyptostroboides* (Arashima et al. 2008; Lee et al. 2009).

## Concluding remarks

The living fossil *M. glyptostroboides*, a tree which seems to have remained unchanged since the Cretaceous era, is a unique source of novel natural products. It is apparent that the chemical defense system of the tree, based on its bioactive secondary metabolites, has resisted the attack of millions of generations of pathogens during geological time. The potential of these compounds and extracts containing them has only very recently been exploited in modern medicine. As a consequence of the significant strides in the development of chromatographic methods and increasingly sensitive spectroscopic instruments, in particular the development of cryogenic probe technology for high-field NMR instruments, discovery of an increasing number of novel natural products from *M. glyptostroboides* is expected to continue in the near future. The fact that several medicinal applications based on compounds from this plant source as active principles currently exist, would encourage such development, including extensive testing of biological activity of these new compounds. The latter point may be further reinforced by the fact that, at present, compounds specific for *M. glyptostroboides* have hitherto only been tested to a limited extent with respect to their biological activity. Indications that the growth and sunlight conditions may significantly influence the qualitative production of the selection of natural products of this species strongly encourage international research cooperation leading to a coordinated global exploitation of plant material from geographically exceptionally different localities.
